# Chromatin loop anchors are associated with genome instability in cancer and recombination hotspots in the germline

**DOI:** 10.1186/s13059-018-1483-4

**Published:** 2018-07-30

**Authors:** Vera B. Kaiser, Colin A. Semple

**Affiliations:** MRC Human Genetics Unit, MRC Institute of Genetics and Molecular Medicine, University of Edinburgh, Western General Hospital, Crewe Road, Edinburgh, EH4 2XU UK

**Keywords:** Cancer, Recombination, DNA breakage, Hi-C, Chromatin loops

## Abstract

**Background:**

Chromatin loops form a basic unit of interphase nuclear organization, with chromatin loop anchor points providing contacts between regulatory regions and promoters. However, the mutational landscape at these anchor points remains under-studied. Here, we describe the unusual patterns of somatic mutations and germline variation associated with loop anchor points and explore the underlying features influencing these patterns.

**Results:**

Analyses of whole genome sequencing datasets reveal that anchor points are strongly depleted for single nucleotide variants (SNVs) in tumours. Despite low SNV rates in their genomic neighbourhood, anchor points emerge as sites of evolutionary innovation, showing enrichment for structural variant (SV) breakpoints and a peak of SNVs at focal CTCF sites within the anchor points. Both CTCF-bound and non-CTCF anchor points harbour an excess of SV breakpoints in multiple tumour types and are prone to double-strand breaks in cell lines. Common fragile sites, which are hotspots for genome instability, also show elevated numbers of intersecting loop anchor points. Recurrently disrupted anchor points are enriched for genes with functions in cell cycle transitions and regions associated with predisposition to cancer. We also discover a novel class of CTCF-bound anchor points which overlap meiotic recombination hotspots and are enriched for the core PRDM9 binding motif, suggesting that the anchor points have been foci for diversity generated during recent human evolution.

**Conclusions:**

We suggest that the unusual chromatin environment at loop anchor points underlies the elevated rates of variation observed, marking them as sites of regulatory importance but also genomic fragility.

**Electronic supplementary material:**

The online version of this article (10.1186/s13059-018-1483-4) contains supplementary material, which is available to authorized users.

## Background

Recent evidence shows that many cancers and developmental disorders involve disruptions of chromatin organisation. Insertions and deletions are reported to alter the boundaries of topologically associating domains (TADs), which normally constrain the regulatory interactions of resident promoters and enhancers, causing dysregulated gene expression [1, 2]. Disruptions of particular TAD boundaries have been reported in neuroblastoma [3, 4], medulloblastoma [5], leukaemia [6, 7] and other cancers [8], consistent with the hypothesis that structural variants (SVs) remodelling TAD boundaries may act as oncogenic ‘driver’ mutations under selection in tumour cells [9].

CTCF plays important roles in chromatin organisation, both demarcating domain boundaries as an insulator element [10, 11] and by bringing DNA sites that are distant in linear genomic distance intro close spatial proximity [12]. According to the loop extrusion model, it is proposed that pairs of CTCF binding sites may physically interact to form anchoring sites at the base of a chromatin loop, acting as physical barriers to the ring-shaped cohesin complex, which slides along the DNA [13–15]. Topological stress relief at loop anchor points may be provided by TOP2B [16, 17], an enzyme that transiently creates double-strand breaks (DSBs) and re-joins the DNA in a different spatial configuration, and TOP2B binding sites in the breast cancer cell line MCF-7 have been shown to be co-located with CTCF motifs [18]. On a larger scale, complex arrays of DNA loops are thought to make up the substructure of regulatory domains such as TADs [19], and recent experiments highlight the critical importance of CTCF for loop and TAD formation [20].

CTCF binding sites are highly mutated across cancer types, especially when they are located within loop anchor points (LAPs) [21, 22]. Hyper-methylation of the GC-rich CTCF binding motif has been shown to reduce CTCF binding in glioma, leading to the up-regulation of known oncogenes [23]. Hnisz et al. [7] have shown that constitutive CTCF–CTCF binding site interactions delineating loops are recurrently deleted in T-cell acute lymphoblastic leukaemia, which leads to oncogene activation. Overall, these data suggest that domain boundary or LAP lesions affecting gene regulation are far from rare in cancers and occur at comparable rates to recurrent in-frame gene fusions [8]. However, it is unclear whether LAPs are intrinsically prone to high mutation rates in cancer, constituting a novel class of fragile sites in the genome, or whether the observed lesions affecting LAPs confer a selective advantage to tumour cells.

Somatic mutation rates vary across the genome, and a large fraction of this variation can be attributed to differences in replication timing, with late replicating regions of the genome accumulating increased levels of single nucleotide variants (SNVs) [24]. Large regions of chromosomes (encompassing hundreds of kilobases) are replicated synchronously in replication domains that correspond closely to TADs, linking chromatin organisation to spatiotemporal variation in replication [25], while other, inter-correlated features of chromatin, such as histone methylation or acetylation patterns, are also associated with somatic mutation rates [26]. On a much finer scale, the individual binding sites of a variety of DNA binding factors, including CTCF, appear to obstruct the lagging strand replication and DNA repair machinery and induce higher mutation rates in human and yeast [27–29]. However, the mutational landscape associated with intermediate levels of chromatin organisation, such as chromatin loops, are not well studied.

Similarly, the influence of genomic features on structural rearrangements in cancer is relatively under-studied, but it appears that different cancer types follow different patterns. For some cancer types, such as breast cancer, structural somatic variants are enriched within early replicating, GC-rich, transcribed regions of the genome, whereas the opposite trend was observed for cancers such as prostate and melanoma [30]. Further, the 3D structure of the genome may predispose regions of the genome that are in Hi-C contact to be more likely to undergo structural rearrangements [31, 32].

A cellular process intrinsically linked to double-strand breakage is genetic recombination, which is involved in DNA repair in the somatic cell and is an essential process in the production of germ cells. Replication- and recombination-associated mechanisms are hypothesised to lead to the formation of structural variants and may, therefore, contribute to structural variation in cancers [33].

During meiosis, recombination is initiated by DSBs and occurs non-randomly across the genome; it is at its highest level at recombination hotspots (HSs) where the majority (60%) of recombination events take place [34, 35]. While it is known that recombination produces large SVs, the effect of recombination on the emergence of SNVs is less clear—as is its relation to chromatin structure. There is evidence that recombination is mutagenic in yeast [36, 37], and a recent study of 283 human trios has shown a correlation between the rate of recombination events in parental germ cell genomes and the rate of de novo SNVs in offspring genomes, suggesting a mutagenic effect of recombination [38]. However, the data supporting this were necessarily sparse, given the low de novo mutation rates in the germline.

Here, we explore the genomic landscape around LAPs and demonstrate that the unusual chromatin environment at LAPs is matched by unexpected mutation rates, establishing LAPs as foci of evolutionary change and fragile sites in cancer.

## Results

**Fig. 1:**
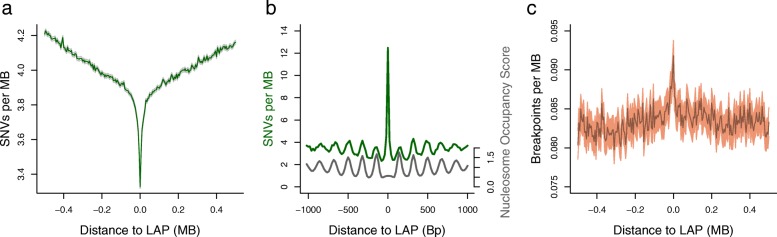
Loop anchor points are sites of genomic instability in cancer. **a** Average SNV rates per tumour within 500 kb of all LAPs. **b** Finer scale average SNV rates per tumour (*green line*) within 1 kb of LAPs, centred on the CTCF site within each LAP, with nucleosome occupancy based on MNase-seq data over the same intervals (*grey line*). **c** Average SV breakpoint rates per tumour within 500 kb of all LAPs. The 95% confidence intervals, calculated assuming a Poisson distribution of mutation rates, are shown as shaded regions in **a** and **c**. Note that the y-axis scaling has been selected to clearly depict statistically significant trends in these data

Previous work has demonstrated elevated SNV rates at CTCF binding sites within LAPs in a variety of cancers [21]. This motivated us to investigate genome-wide somatic mutation rates around CTCF-containing LAPs from the aggregated Hi-C datasets of Rao et al. [19] (see “Methods”), using 13 recently released ICGC somatic variant datasets ascertaining both SNVs and SVs in nine different tumour types [39]. Within 50 kb of LAPs, the ICGC pan-cancer samples show a dramatic drop in SNV rates (Fig. 1a). This regional decrease in SNVs at LAPs is in stark contrast to the high mutation rate observed at the short 19-bp CTCF-binding motifs located inside LAPs, which is 12.3 SNVs/Mb^− 1^, or more than three times higher than the local background mutation rate. Plotting SNV rates at 20-bp resolution, a peak of SNVs in the centre of the LAPs at CTCF-binding sites becomes apparent (as seen in Kaiser et al. [21]; Fig. 1b and Additional file 1: Figure S1), as well as a periodic pattern of mutation reflecting nucleosome occupancy [28, 40]. Thus, CTCF-binding sites within LAPs are prone to local somatic hypermutation in tumours but often reside within broader genomic regions with significantly reduced SNV rates.

### Chromatin loop anchors are HSs of structural variation in tumours

**Fig. 2:**
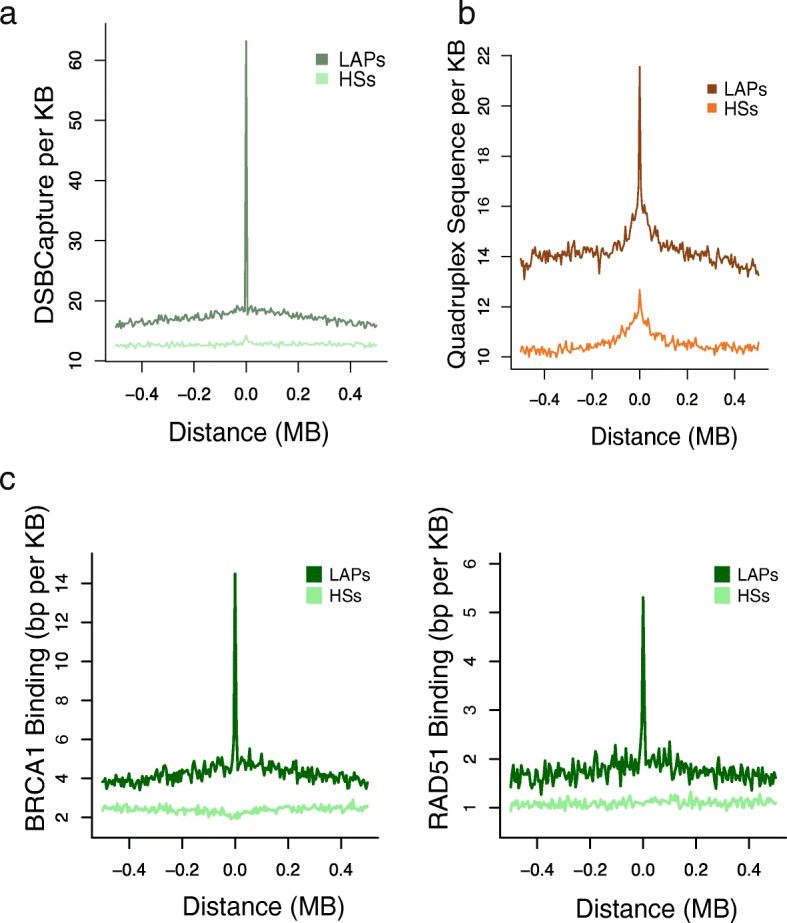
Loop anchor points are susceptible to somatic double-strand breaks. **a** DNA double-strand break density in the NHEK cell line, within 500 kb of NHEK LAPs and recombination HSs. **b** Predicted quadruplex structure forming sequence density within 500 kb of the union set of LAPs. **c** Average density of BRCA1 ChIP-seq binding peaks for MCF-10A cells and RAD51 ChIP-seq binding peaks for MCF-7 near HMEC LAPs and recombination HSs

**Fig. 3:**
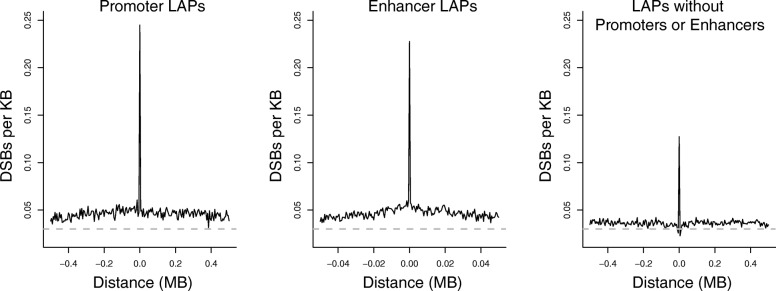
Rates of spontaneous DSBs increase at LAPs, also when no promoter or enhancer sequence is present. The overlap, per kilobase, with NHEK DSB regions is shown for NHEK LAPs that overlap the following ENCODE chromHMM annotations: NHEK promoters (762 LAPs), NHEK enhancers (1485 LAPs), or neither NHEK promoters or NHEK enhancers (2030 LAPs). The *dashed line* indicates the genome-wide average

In contrast to SNVs, the frequency of pan-cancer SV breakpoints shows a significant increase at LAPs, inverting the pattern seen for SNVs over the same range of flanking sequence (Fig. 1c). This shows that LAPs are structurally fragile sites in cancer, and so we examined associations between LAPs and more direct measures of genomic instability. Lensing et al. [41] identified genome-wide foci of endogenous DSBs in vitro and these sites show a striking ~ 3.7-fold enrichment at LAPs compared to their flanking regions (Fig. 2a). A proportion of this enrichment may be attributable to the close proximity of LAPs to promoters and enhancers, which are known to suffer elevated DSB rates [41]. However, LAPs lacking any overlap with known promoters and enhancers show similarly elevated rates to those that do (Fig. 3). Consistent with inherent genomic instability, LAPs are also enriched in predicted G-quadruplexes (G4s), a DNA secondary structure associated with regulatory regions and DSB formation in cancers [42] (Fig. 2b).

BRCA1 and BRCA2 are two well-characterised tumour suppressor genes involved in DSB repair by homologous recombination [43–45]. BRCA1 is often recruited to sites of active transcription, which are prone to DNA damage during the formation of transcriptional R-loops [46]. We found a strong enrichment of BRCA1 at HMEC LAPs in MCF10A, a normal breast epithelial cell line, as well as increased RAD51 binding—which mediates BRCA2 binding—around LAPs in the MCF-7 cell line (Fig. 2c); this is, to our knowledge, the first observation of BRCA1/2 association with LAPs, albeit this appears to be driven by neighbouring active promoters and enhancers rather than the LAPs themselves (Additional file 1: Table S1).

### LAPs overlap recombination HSs in human populations

**Fig. 4:**
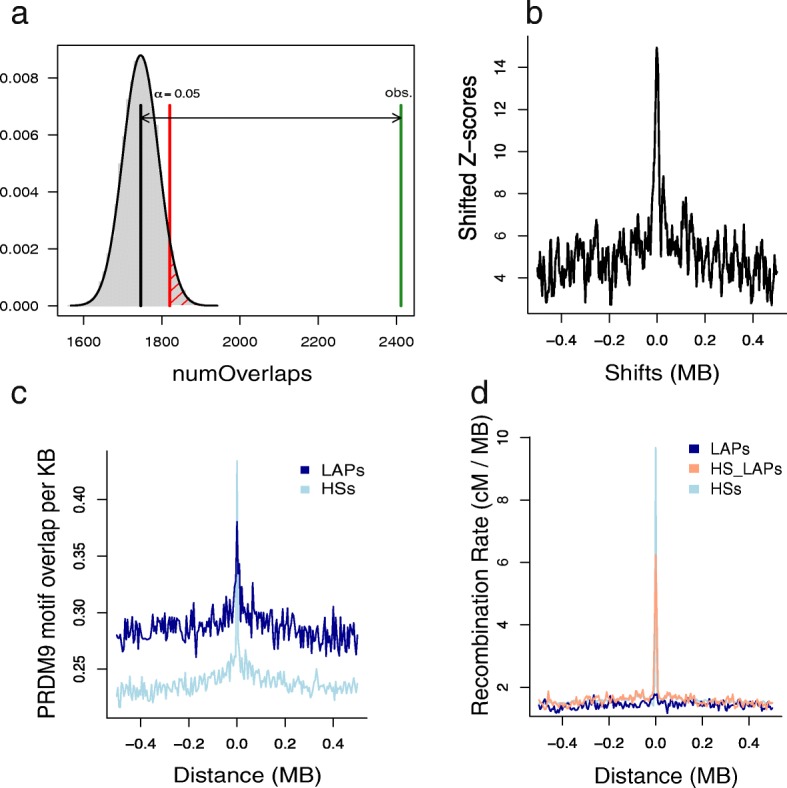
LAPs and recombination HSs are physically linked. **a** Probability density plot showing the observed number (*green line*) versus expected distribution (*grey histogram*) of overlaps between LAPs and HSs, *p* < 10^− 5^ based upon 100,000 circular permutations. The threshold for statistical significance is indicated by the *red line*. **b** Variation in circular permutation z-scores (y-axis) relative to shifts in the location of HSs (x-axis), suggesting that significance of overlaps between LAPs and HSs results from specific local (not broader regional) overlaps. **c** PRDM9 binding motif density, which is targeted by the recombination machinery, within 500 kb of recombination HSs and LAPs. **d** Average recombination rates within 500 kb of recombination HSs, the subset of LAPs overlapping HSs (HS_LAPs) and all LAPs

Intriguingly, LAPs show an unexpected genome-wide correspondence with germline recombination HSs, calculated from genotyping of extant human populations [47], such that 16% of LAPs overlap HSs (based upon 100,000 circular permutations; *p* <  10^− 5^; Fig. 4a). These overlaps are notably precise, so that the association between LAPs and HSs drops when the two sets of regions are shifted with respect to each other by less than 50 kb (Fig. 4b). Thus, their correspondence is not simply attributable to the enrichment of both sets of features within certain broader neighbourhoods, such as replication timing domains or nuclear compartments. Recombination HSs are known to often contain the motif bound by PRDM9, a critical component of the recombination machinery [48, 49], and, using stringent search criteria (see “Methods”), we find this motif in 17% of HSs. Similarly, we find that 13% of LAPs also contain at least one PRDM9 core motif, which is an enrichment of ~ 33% compared to the median number of motifs per 5-kb bin in LAP flanking regions (Fig. 4c) and also constitutes a significant enrichment genome-wide (circular permutations in *R*: *p* < 10^− 5^). For the 16% of LAPs directly overlapping HSs (HS-LAPs) we find no further enrichment of the PRDM9 motif (13.6% of HS-LAPs contain the motif), but, as expected, there is a notable increase in the recombination rate at HS-LAPs (Fig. 4d). This is consistent with dual roles for a subset of LAPs, both as units of chromatin organisation and as HSs of structural variation.

**Fig. 5:**
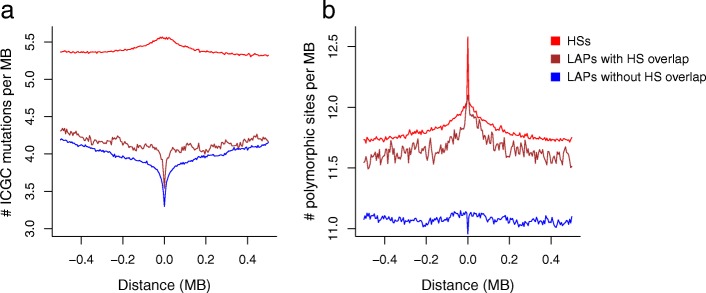
The impact of loop anchor points on tumour and population variation. **a** Average SNV somatic mutations based on ICGC pan-cancer tumour sequencing data and **b** the average number of polymorphic segregating sites within 500 kb of LAPs and HSs. LAPs are subdivided into those that do or do not overlap recombination HSs, respectively

The recombination enzyme PRDM9 is expressed exclusively in testis, but it is also expressed in a variety of cancer cell lines and samples and has been proposed as a cancer biomarker [50]. We observe modestly increased SNV rates at recombination HSs in cancer (Fig. 5a) but do not find any pan-cancer increase in SV breakpoints around HSs (Additional file 1: Figure S2), which might be expected if meiotic recombination complexes were activated in the tumours examined here. In addition, the histone modification H3K4me3, which is deposited by PRDM9 at DSBs, is not observed at recombination HSs in the cancer cell lines HepG2 and MCF-7 (Additional file 1: Figure S3). In contrast, H3K4me3 increases around LAPs (Additional file 1: Figure S3), possibly as a result of PRDM9 recruitment to the PRDM9 motifs enriched at LAPs or, more likely, because H3K4me3 is a mark of active promoters enriched at chromatin boundaries [11]. We cannot, however, exclude the possibility that PRDM9 is active in at least a subset of the tumours under investigation and contributes to the increase in SV rates at LAPs.

A substantial fraction of LAPs (47% of those studied here) constitute regulatory domain boundaries [19], while an even higher proportion, 69%, overlap DSB-foci [41], and 16% overlap recombination HSs (Table 1). Genome-wide, however, these three categories of LAPs appear to be largely independent, as the extent of overlap between categories was remarkably similar to the expected rate assuming independent distributions across the genome. For example, LAPs that appear as domain boundaries were as likely to overlap recombination HSs as LAPs that do not act as boundaries (Table 1). There was also no enrichment of Gene Ontology (GO) terms associated with genes neighbouring HS-LAPs versus a background set of genes found at all LAPs, i.e. HS-LAPs are not found near specific functional categories of genes.

**Table 1:** Overlap of LAPs with domain boundaries, recombination HSs and DSB-prone regions

a) Feature	Feature count	Genomic span (Mb)		
LAPs	14,737	73.69		
Boundaries	40,615	250.89		
HSs	32,984	181.79		
DSB-prone regions	84,946	34.69		
b) Intersection	Number of LAPs	Fraction of LAPs	Expected fraction (circular permutations)	*p* value
LAPs ∩ Boundaries	6960	0.47	0.15	< 10^−4^
LAPs ∩ HSs	2385	0.16	0.12	< 10^−4^
LAPs ∩ DSB-prone regions	10,133	0.69	0.14	< 10^−4^
c) Intersection	Number of LAPs	Fraction of LAPs	Expected fraction (feature intersection)	*p* value
LAPs ∩ HSs ∩ DSB-prone regions	1709	0.12	0.11	< 0.001
LAPs ∩ HSs ∩ boundaries	1172	0.08	0.08	< 0.05
LAPs ∩ HSs ∩ boundaries ∩ DSB-prone regions	836	0.06	0.05	< 0.05

**a** The number of features and their genomic span. **b** The number of LAPs that overlap with domain boundaries, HSs or DSB-prone regions, as well as the fraction of unique LAPs in each intersection (as a proportion of the total dataset of 14,737 LAPs). The expected fraction (*column 4*) and the corresponding *p* value (*column 5*) were calculated based on 10,000 circular permutations in *R*. **c** *Columns 2* and *3* as in **b**. The expected fraction (*column 4*) was calculated by multiplying the respective fractions shown in *column 3* of **b**. For example, the fraction of LAPs in the intersection “LAPs ∩ HSs ∩ DSB-prone regions” is equal to 0.16 × 0.69 = 0.11. The hypergeometric test was used to assess the statistical significance of overlap (*column 5*)

LAPs are associated with similar mutational landscapes in tumours, irrespective of whether they overlap HSs or locate outside high recombination regions: both types of LAP show a distinctive dip in tumour SNV rates (Fig. 5a), while cancer mutation rates somewhat increase around recombination HSs, by ~ 3.5% compared to the median rate within the flanking regions. As expected, recombination HSs are associated with a pronounced increase in SNPs in the 1000 Genomes Project (1KG) dataset [47], and LAPs that overlap HSs are also enriched for segregating variants, by ~ 7% compared to the flanking regions (Fig. 5b). Accordingly, population genetic processes that increase variation at HSs—such as selective sweeps and reductions in background selection [51]—also appear to have impacted germline variation at HS-LAPs. However, germline de novo SNV rates at LAPs are not reduced as they are in cancer and, similarly, we do not observe an increase in SVs near HSs in cancer (Additional file 1: Figure S2), suggesting fundamentally different influences on germline mutation rates versus cancer-associated somatic mutation rates at these sites.

### Cancer mutation rates at CTCF sites outside LAPs and at non-CTCF LAPs

**Fig. 6:**
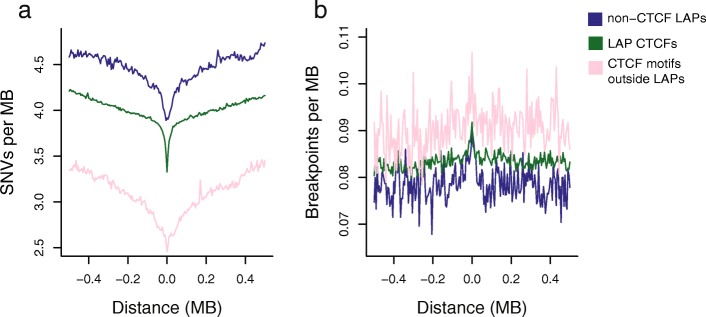
Somatic mutation rates around CTCF-containing LAPs, non-CTCF LAPs and CTCF sites outside the known set of LAPs. **a** SNV rates; **b** DSB rates. The central 5-kb windows of all three plots in **b** are statistically elevated compared to the flanking regions (resampling one million times with replacement; *p* <  0.01)

To investigate if the observed mutational patterns at LAPs are due to the presence of CTCF binding alone, we also studied mutational patterns around constitutive CTCF-binding sites located outside the known set of LAPs, i.e. sites that may not act as anchor points. Compared to the CTCF-LAPs, we observe a similar decrease of SNV rates in the 1-Mb sequence surrounding CTCF sites (Fig. 6) and a less distinct peak of mutation when zooming in to high resolution (Additional file 1: Figure S4). However, we note that an unknown proportion of these CTCF sites may also be involved in loop formation but were not detected in present LAP data—either because they participate in transient or cell type-specific LAPs or because the sequencing depth was insufficient in the original cell lines. Indeed, the available data suggest that LAP detection is a linear function of Hi-C sequencing depth and many loops remain to be discovered (Additional file 1: Figure S5). Non-LAP CTCF sites also showed an increased rate of DNA breakage (Fig. 6b) and a strong enrichment of BRCA1 (Additional file 1: Figure S4), consistent with general co-binding of these factors and recent data which suggest that CTCF has roles in regulating the homologous recombination repair pathway [52], as well as a high degree of genome fragility for these non-LAP CTCF sites.

Conversely, we considered a stringent set of 2102 LAPs that are devoid of CTCF motifs, with no evidence for CTCF ChIP-seq binding across ENCODE datasets. With respect to mutation rates, these non-CTCF LAPs behave similarly to CTCF-containing LAPs and contain similar mutational signatures to CTCF-LAPs (Fig. 6 and Additional file 1: Figure S6), while CTCF motifs inside CTCF-LAPs showed no evidence for specific mutational signatures associated with processes such as APOBEC editing after single-strand DNA exposure [53, 54] (Additional file 1: Figure S7). This suggests that the genome architecture or other common features of LAPs impact on their propensity to mutation, such as the presence of enhancer-promoter loops [16].

The most common motifs at non-CTCF LAPs were the promoter-associated motifs MA0506.1 (NRF1), MA0516.1 (SP2), and MA0079.3 (SP1), but none of these motifs showed any enrichment compared to CTCF-binding LAPs. Interestingly, the overlap between recombination HSs and LAPs is confined to LAPs that bind CTCF and is not found for the stringently defined set of 2102 non-CTCF LAPs. Only 11% of non-CTCF LAPs overlap HSs (not significant by circular permutations) and only 5% contain the PRDM9 motif, which is a significant depletion compared to expectation (*p* = 0.0044).

### Chromatin features influence increased mutation rates at LAPs

LAPs are relatively GC-rich, enriched for histone modifications associated with active transcription (H3K27ac, H3K27me3, H3K36me3, H3K4me3 and DNase sensitive open chromatin) and depleted for the repressive mark H3K9me3; they also tend to be found in relatively early replicating regions of the genome (Additional file 1: Figure S3). Recombination HSs also show locally increased GC content, but otherwise possess a contrasting set of features, consistent with their presence in later replicating regions (Additional file 1: Figure S3). Accordingly, LAPs tend to be enriched for genes and actively transcribed regions [19], whereas HSs are located, on average, further away from genic sequence. This raises the possibility that the unusual mutational properties of LAPs may be explained by their distinctive chromatin and sequence features.

To investigate this further, we first identified the specific tumour type most suited for an in-depth analysis. Additional file 1: Figure S8 shows SNV and SV rates around LAPs for all nine cancer types in this study separately. SNV rates are consistently reduced near LAPs in several tumour types, but there are exceptions, such as the malignant lymphoma (MALY-DE) dataset, which does not show a pronounced dip in mutation rates near LAPs even though it includes a large number of SNVs (Additional file 1: Table S2). A pan-cancer analysis is best suited to highlight general patterns, but, for SVs in particular, stratifying mutations by tumour type reduces the power to detect any patterns on a per-tumour basis as SVs are, on average, ~ 100-fold less frequent than SNVs (Additional file 1: Table S2). Beyond differences in dataset sample sizes, the variability among tumour types most likely reflects the limitations of the current Hi-C data, which may be poorly matched to the cells in some cancer samples. However, we could make use of the high-resolution mammary epithelium LAPs from the Rao et al. [19] dataset, which are well matched to the breast cancer data, and a range of epigenetic information is available for this tissue type; note that breast cancer mutations showed both a clear dip in SNV rates and a peak of SV rates near LAPs (Additional file 1: Figure S8).

**Fig. 7:**
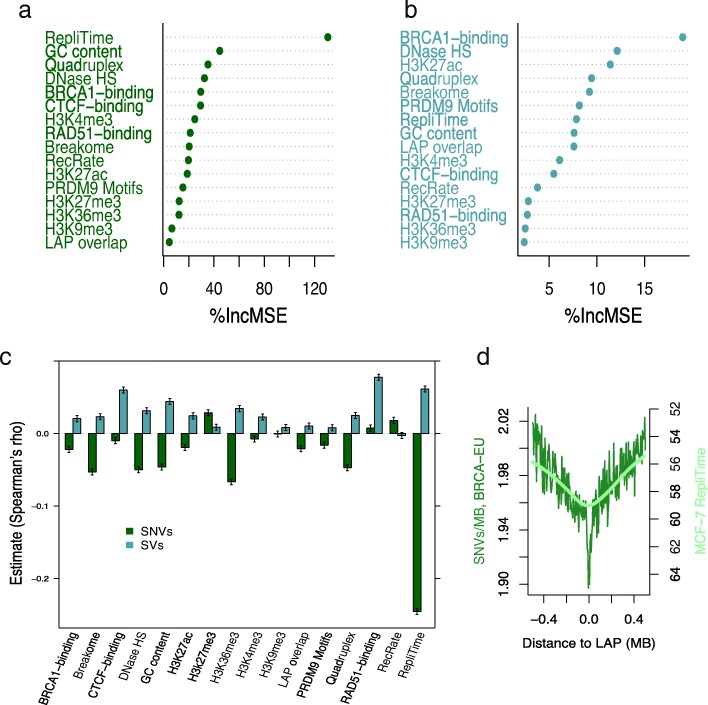
Genomic and epigenomic features influencing SNV and SV rates at loop anchor points in breast tumours. **a, b** Variable importance, measured as percentage increase in mean squared error (%IncMSE) when each predictor variable is removed from the model, predicting SNV rates (**a**) or SV breakpoint rates (**b**), respectively. **c** Spearman’s rank correlation coefficient estimates between mutation rates and predictor variables of the random forest models, for SNV rates (*green*) and SV breakpoint rates (*blue*); 95% confidence intervals of the estimates are indicated. **d** SNV rates in BRCA-EU breast tumours within 500 kb of LAPs in the MCF-7 breast tumour cell line relative to replication timing (average MCF-7 ENCODE Repli-seq signal); higher Repli-seq values indicate earlier replication time

Accordingly, we assessed the extent to which mammary epithelium-derived chromatin features (from the MCF-7 and MCF10A cell lines) and a variety of other cell type-specific features were associated with mutation rates in a large ICGC breast tumour dataset (BRCA-EU). Specifically, we used random forest regression to construct models of mutation rates observed within all 5-kb windows from the 500-kb regions flanking all mammary epithelium LAPs (derived from HMEC cell line Hi-C data) plus the 5-kb LAP regions themselves (“Methods”). Similar models have previously shown high predictive accuracy in modelling aspects of nuclear organisation and provide variable importance estimates that are robust to the inter-correlated nature of chromatin feature input variables [11]. In our model, by far the most important predictor of the BRCA-EU SNV rate was replication timing, with reduced levels of mutation observed in early replicating regions (Fig. 7), consistent with other studies of breast cancer mutation patterns [40]. The correlation coefficient between observed and predicted SNV rates from the random forest model (*r* = 0.28; *p* value < 10^− 15^) suggests a significant influence of the features included but, overall, a moderate level of predictive accuracy. Modelling was less successful in predicting BRCA-EU SV breakpoint rates (*r* = 0.09 between observed and predicted values) but also indicated a significant association with chromatin and sequence features (*p* value < 10^− 15^) (Fig. 7b). However, even though the magnitude of effects is rather modest, the direction of associations is strikingly inverted for SNV and SV rates, such that the variables most strongly correlated with elevated SV rates (DNaseHS, replication timing, G-quadruplex content, GC content) are associated with decreased SNV rates (Fig. 7c). We conclude that similar chromatin and sequence features have moderate, but largely opposing, effects upon SNV and SV rates at LAPs.

Cell type-specific features are not available for most of the cancer types studied, but we carried out random forest regression analysis on mutation rates in each of the nine tumour types separately, using, as input features, only cell type-invariant features around the union set of 14,737 LAPs (Additional file 1: Table S3). As expected, the previously reported associations between mutation rates and replication timing, GC content and quadruplex sequence are seen across all tumour types. The estimates of the influence of overlapping LAPs (the variable “LAPs” in Additional file 1: Table S3) on mutation rates directly reflects the dip—or lack thereof—of mutation rates at LAPs seen in a given tumour type (Additional file 1: Figure S8). For example, in the case of melanoma, where a strong decline in SNV rates is evident, this variable influences mutation rates strongly as reflected in a high %MSE score. In contrast, in malignant lymphoma, where the SNV decline at LAPs is very modest, LAP overlap has little measurable effect on SNV rates.

### Genes within recurrently disrupted chromatin loops are enriched for functions in the cell cycle

Recent literature has documented tumours showing oncogene upregulation as a result of disrupted CTCF binding sites and chromatin loops in a variety of cancers [1]. Using breakpoint data from all 1672 ICGC donors and the union set of 14,737 CTCF-containing LAPs, we find that genes within the top 5% most disrupted chromatin loops (possessing five or more SV breakpoints in their LAPs) are enriched for functional annotation terms associated with proliferation and the G1/S cell cycle transition (Additional file 1: Table S4) [55]. The enrichments of such putatively cancer-associated terms are often only marginally significant given current sample sizes, but are broadly consistent with previously reported oncogenic disruptions [1]. Thus, it is possible that frequent disruptions of chromatin loops and domain boundaries in proximity to oncogenes in tumours are driven by the unusual mutational biases at LAPs.

In the breast cancer dataset, we also observe an unexpected excess of overlap between recurrently disrupted HMEC loops (bordered by LAPs that are disrupted in at least two samples) and GWAS regions associated with breast cancer: there were 40 such overlaps, whereas only 18.1 overlaps were, on average, observed in 5000 circular permutations (*p* = 0.0002). This excess in overlap is notably larger than that observed for the background set of all HMEC loops and GWAS hits (225 observed overlaps and a mean of 143.6 expected overlaps, based on 5000 permutations; *p* = 0.001), suggesting a possible causal relationship between LAP disruption and the breast cancer phenotype.

#### Common fragile sites and LAPs

Common fragile sites (CFSs) are large, initially cytogenetically defined, genomic regions characterized by high rates of DNA breakage, active transcription and late replication [56, 57]. Interestingly, we found more LAP–gene intersections for protein-coding genes within CFSs compared to LAP–gene intersections in the genome as a whole (0.77 and 0.47 LAPs per gene; relative proportion test in R *p* < 10^− 6^), while the average replication timing was indistinguishable between the two sets of LAPs (Wilcoxon test in *R*, not significant). If genes are more often interrupted by LAPs in fragile regions, this leaves the intriguing possibility that at least a subset of CFSs are caused by DNA looping and the instability associated with LAPs.

## Discussion

LAPs and recombination HSs are two seemingly unrelated features of the genome—one involved in chromatin organisation and the other in recombination during meiosis—but both classes of sites emerge as hotspots for DSBs. We have shown that LAPs and HSs often occur in the same genomic locations, which suggests that the same genomic regions that migrate to the chromosomal axis during meiosis, ultimately forming the points of breakage for DSB initiation [58], are also involved in chromatin organisation in the interphase nucleus of somatic cells. Intriguingly, cohesin, which associates with CTCF at LAPs [59, 60], is also enriched at the meiotic loop axis and plays a diverse role in chromosome pairing in both mitosis and meiosis [61]. Coincidentally, a recent study has shown that a subset of PRDM9 binding occurs at CTCF sites in mouse spermatocytes, and an interaction between the two proteins has been suggested [62]. This observation is consistent with an enrichment of recombination HSs at CTCF- LAPs (but not at non-CTCF LAPs), while the enrichment of PRDM9 motifs within CTCF-LAPs also suggests a sequence-based mechanism. A variety of factors, many related to chromatin structure, affect the propensity of LAPs to harbour SV breakpoints. PRDM9 also appears to be active in at least a subset of cancer cells [50] and may contribute to DSB formation at LAPs, suggesting another possible link between LAPs and HSs. Further, the association of LAPs with DSB formation appears to be at least partly attributable to the enrichment of active promoters and enhancers at LAPs, which is consistent with reports that promoters are inherently prone to DSBs, in both somatic cells [41] and meiotic cells that lack PRDM9 [63]. However, we also observe an excess of DSBs at LAPs that have no overlap with promoter or enhancer states, demonstrating that DNA breakage is not solely due to these elements. LAPs are unusual with respect to replication timing, with LAPs replicating, on average, earlier than their surrounding regions, consistent with the dual roles of cohesin in stabilising chromatin loops and also initiating replication [64]. Accordingly, chromatin looping may, to some extent, directly result from the initiation of replication or, conversely, determine its starting position in the next cell cycle [65]. Given the strong association between LAPs and DSB-prone regions, disruption of replication near such origins may be one way in which genome instability is introduced in cancer [66]. Notably, regions stably bound by DNA binding proteins such as CTCF seem to suffer high mutational loads due to replication errors [27].

LAPs are foci of DSB breakpoints and may provide the raw material for cancer evolution via structural variation, dependent on other factors, such as deficiencies in DNA repair pathways. The resulting SVs may have been subject to selection in cancer, though the majority are likely to be ‘passenger’ variants that drift toward fixation with little phenotypic consequences in their tissue of origin. Accordingly, we observe a strong enrichment of somatic DSB formation at LAPs in the NHEK cell line, more modest elevations in SV breakpoints around LAPs in cancers, and only some evidence that the genes affected are those that experience selection in cancers. At the human population level, our results suggest that chromatin loops are predominantly inherited as a genetic unit, with recombination often confined to LAPs, and therefore tending to preserve regulatory haplotypes. Consistent with our results, recent studies have shown that linkage disequilibrium (LD) blocks are enriched within TADs, i.e. recombination between enhancers and their target genes is reduced within domains [67]. Indeed, HSs themselves may primarily be a by-product of particular chromatin environments, replication timing and other functional constraints, such as a lack of active transcription, which may interfere with the recombination process [68].

We have used aggregate analysis across loop anchor points and cancer types to show that a mutational bias towards somatic breakage of chromatin loop anchors exists, consistent with recent experimental data from mouse B cells [16]; notably, breakage is more prominent in some cancer types than others and presumably depends on the general genome instability of the tumour type. The unusual DNA breakage patterns near LAPs are likely to contribute to cancer evolution, reflected in higher SV breakpoint levels, and allowing for novel promoter–enhancer interactions. The increase in breakage near LAPs is influenced by their specific chromatin environment and replication timing, DNA folding and accessibility to the repair machinery. Similar influences may underlie the surprising association of LAPs with meiotic recombination events.

## Conclusions

In this study, we show that chromatin loop anchor points are fragile sites in the genome, acquiring DNA breakage in a range of cellular contexts, such as in normal cell lines, in cancer and during meiosis. The implications of this are far-reaching, affecting gene regulation in somatic tissues as well as the modular structure of the genome during evolution.

## Methods

### LAP and recombination HS datasets

Chromatin loops for cell lines representing all human germ layers (GEO dataset GM12878, HeLa, HMEC, HUVEC, IMR90, K562 and NHEK) were derived from unusually high resolution in situ Hi-C data, defining LAPs at a resolution of 1–5 kb (accession GSE63525) [19]. These loops are often conserved between cell lines, such that 55–75% of the loops detected in any given cell line were also found in the most deeply sequenced cell line (GM12878), and around 50% appear to be conserved across mammalian species [19]. The majority of loops are also associated with convergently orientated CTCF binding motifs at the putative LAPs, consistent with the known roles of CTCF in loop formation [19]. On average, 17% of LAPs were only observed in one tissue (Additional file 1: Figure S5), with more deeply sequenced cell lines consistently resulting in more LAPs being called. From this dataset, we created a merged dataset of 14,737 LAPs, centred around their associated (and convergently orientated) CTCF motifs [19], which represents the union of LAPs across all cell lines. For this purpose, the seven files annotated as “looplist_with_motif” for human cell lines were downloaded from the GEO dataset GSE63525; the genomic locations of CTCF motifs assigned to LAPs were merged if overlapping, such that each motif was only counted once, and the flanking sequence extended to 5 kb. This merged dataset was used for all analyses except for the breast cancer-specific analysis, where we used only LAPs derived from the HMEC (human mammary epithelial cells) Hi-C data, and the DSB analysis, where tissue-matched NHEK (normal human epidermal keratinocyte) LAPs were used, both of which were provided by [19].

To establish a control set of CTCF sites that were presumably *not* acting as loop anchors, we intersected CTCF motifs found in constitutively open chromatin [21] with all LAPs of the Rao et al. [19] dataset (plus 10 kb of flanking region), which resulted in 1845 constitutively bound CTCF motifs that were not found near LAPs, 1300 of which were in a uniquely mappable sequence context. We want to highlight, however, that this dataset may contain many false negatives, i.e. CTCF sites that act as anchor points in undetected LAPs.

Conversely, we used a union set of 11,890 merged LAPs for which a localized CTCF motif could not be found as control non-CTCF LAPs. However, since Rao et al. [19] had used very stringent search criteria for CTCF anchor sites, such as requiring SMC3/RAD21 binding, we conservatively removed from this dataset 9539 LAPs in which we detected a CTCF motif, using default search parameters in FIMO [69]. A further 249 of the remaining LAPs were bound by CTCF in encode datasets [70], leaving a total of 2102 conservatively called non-CTCF LAPs, 1578 of which were in a uniquely mappable sequence context. The sets of non-LAP CTCF sites and non-CTCF LAPs were used to create Additional file 1: Figure S4. To test for an enrichment of other transcription factor binding motifs at non-CTCF LAPs, we intersected the genomic coordinates of these LAPs with the locations of 118 motifs found in constitutively open chromatin [21].

Recombination hotspot locations had been identified in the phase II HapMap dataset (release 21) [71, 72]; recombination rates and SNP data were derived from phase 3 of the 1000 Genomes Project [47]. For the mutation rate analysis, we created 1-Mb windows around the midpoints of LAPs and HSs and omitted regions containing ENCODE blacklisted genomic regions (http://hgdownload.cse.ucsc.edu/goldenPath/hg19/encodeDCC/wgEncodeMapability), resulting in 11,085 union LAPs, 6214 HMEC LAPs, and 18,914 recombination HSs plus their respective flanking regions. Median distances between the centre points of adjacent features were 106,331 bp for union LAPs, 125,000 bp for HMEC LAPs and 55,500 bp for HSs, respectively. We divided each 1-Mb region around a LAP into 5-kb non-overlapping windows; measures of mutation rates and overlap with other genomic features were calculated for each 5-kb bin.

### Cancer mutation rates

Release_23 ICGC (https://dcc.icgc.org/) datasets with whole-genome sequence-derived calls for both SNV and SVs (not under publication moratorium in April 2017) were included: BOCA-FR (bone cancer), BRCA-EU (breast cancer), CLLE-ES (chronic lymphocytic leukemia), LIRI-JP (liver cancer), MALY-DE (malignant lymphoma), MELA-AU (skin cancer), OV-AU (ovarian cancer), PACA-AU (pancreatic cancer), PAEN-AU (pancreatic cancer), PAEN-IT (pancreatic cancer), EOPC-DE (early onset prostate cancer), PRAD-CA (prostate cancer), PRAD-UK (prostate cancer). The combined analysis included a total of 32,105,808 SNVs and 368,480 structural variants (Additional file 1: Table S1). We included all categories of structural variants that were listed in the ICGC files (i.e. insertions, deletions, inversions etc.), recording all breakpoint positions based on the coordinates of the SVs (i.e. a single SV has two breakpoint positions). SNV and breakpoint rates were intersected with the genomic coordinates of LAPs and HSs. Confidence intervals (as shown in Fig. 1) were calculated based on the assumption that breakpoint and SNV rates are random processes and follow the Poisson distribution: for this purpose, the aggregate number of mutations over all samples and 5-kb windows was calculated. This number is of the order of half a million for SNVs and ~ 8000 for SVs. For example, a 5-kb window with ~ 4 SNVs per ICGC sample per megabase contains a total aggregate number of 506,363 SNVs (4 SNVs × 5/1000 × 2284 samples × 11,085, the number of times a window was sampled). The corresponding 95% confidence interval for the aggregate number of SNVs was calculated using the poisson.test() function in R, in the example as [504,970, 507,760].

The *R* package SomaticSignatures [73] was used to calculate mutational signatures at union CTCF-LAPs, non-CTCF LAPS and CTCF motifs for each tissue type separately.

### Genomic features near LAPs and recombination HSs

Germline de novo mutation rates were reported for a whole genome sequencing study of 283 Icelandic trios [38]. A range of genomic features were generated by the ENCODE consortium [74], including average replication timing for each 5-kb genomic window, which was calculated based on the Repli-seq wavelet-smoothed signal for MCF-7 (breast cancer) and HepG2 (liver cancer) cell lines; open chromatin sites (DNAse hypersensitivity), CTCF-binding in MCF-7; RAD51-binding in MCF-7; Broad chromHMM tracks for chromatin state segmentation of HMEC; histone modifications (H3K27ac, H3K09me3, H3K4me3, H3K36me3 and H3K27me3) in MCF-7 and HepG2; nucleosome occupancy scores for GM12878. Genomic features based on ChIP-seq data were represented as genomic segments (peaks called) in the ENCODE distributed files; the overlap (in base pairs) between these features and genomic windows around LAPs was calculated using bedtools [75]. GC content for each 5-kb window was also calculated using bedtools [75]. Sites predicted to adopt quadruplex conformations were generated by Kudlicki [76] (http://moment.utmb.edu/allquads/). DSBs were detected using the DSBCapture protocol in the NHEK cell line (GEO database accession GSE78172) [41], and, as in Lensing et al. [41], we used the intersection of both biological replicates as a high confidence DSB peak set. ChIP-seq data for BRCA1 binding in MCF-10A cells were generated by Gardini et al. [77] and MACS2 [78] was used to call peaks in BRCA1 binding using default parameters.

Average replication time was calculated from the Wavelet-smoothed Signals of the 15 Encode cell types available at the UCSC Genome Browser site (http://genome.ucsc.edu/).

### Random forest regression analysis of mutation rates

Random forest regression models were constructed using the *R* package randomForest [79]. To construct a model with 200 trees, we extracted genomic regions within 1 Mb of a HMEC LAP, merging overlapping regions, i.e. counting each unique genomic region once. Response variables were the number of BRCA-EU SNVs or SVs per 5-kb window, for a total of 239,141 windows. Predictor variables were replication timing in MCF-7; GC content; quadruplex overlap; HapMap recombination rate; DSB regions in NHEK cells; MCF-7 DNAse hypersensitivity; BRCA1-binding in MCF-10A; RAD51-binding in MCF-7; CTCF-binding in MCF-7; PRDM9 motif coverage; overlap with peaks of H3K4me3, H3K27ac, H3K36me3, H3K27me3 and H3K9me3 for MCF-7 and MCF-10A cell lines; HMEC LAP presence.

### Random forest regression analysis of BRCA1/2 binding

To model the outcome variables ‘BRCA1 binding’ and ‘RAD51 binding’ in MCF-10A and MCF-7 cell lines, respectively, we divided the genome up into 5-kb windows around 1 Mb of HMEC LAPs. Input features to the model were all 15 Broad chromHMM states for state segmentation in HMEC cells as well as the overlap with HMEC LAPs. Two-hundred random forest trees were constructed.

### LAP and recombination HS overlap

Circular permutation within the *R* package RegioneR [80] was used to assess the significance of genome-wide overlap between LAPs and recombination HS, using 100,000 permutations. The FIMO algorithm [69] from the MEME package [81] was used to scan the genome for occurrences of the 13-bp PRDM9 motif CCTCCCTNNCCAC, using default parameters; this resulted in 51,107 motif locations being identified with a motif match *p* value < 1.3e-06.

### Functional enrichment analysis of recurrently disrupted LAPs

Functional annotation enrichment analysis was carried out for regions of interest using the GREAT tool to calculate FDR-corrected hypergeometric q-values for the default selection of annotation ontologies [82]. As the background set for enrichment analyses, we used the 9973 genomic regions encompassed by all loops within the union set of LAPs. As the foreground set, we used 398 genomic regions encompassed by LAPs disrupted five or more times across tumour samples, corresponding to the top 5% most disrupted loops.

Breast cancer associated SNPs were obtained from the GWAS catalogue [83] (2017-05-29 release) and their coordinates were extended by 5 kb (to account for LD tagging of nearby causal SNPs) according to the average span of LD blocks in 1000 Genomes Project data for European populations [84]. The resulting GWAS SNP-containing segments were merged using bedtools [75] to create a non-redundant set of GWAS regions; circular permutations were carried out in *R* to test for an excess of overlap between the GWAS regions and chromatin loops in the HMEC cell line.

### Common fragile sites dataset

The genomic locations of 70 protein-coding genes within CFSs (annotated as “common fragile sites”) were downloaded from the NCBI Gene database.

### Programming languages

Datasets were downloaded and formatted using unix shell scripting. Manuscript figures were created using custom scripts in *R* [85].

## Additional files

Additional file 1: Chromatin loop anchors are associated with genome instability in cancer and recombination hotspots in the germline. **Figures S1–S8** and **Tables S1–S4.** (PDF 641 kb)

Additional file 2: Review history of this manuscript. (DOCX 199 kb)
