# Supplementary material for: Chromatin loop anchors are associated with genome instability in cancer and recombination hotspots in the germline
Source: Genome Biol. 2018 Jul 30;19:101. doi: 10.1186/s13059-018-1483-4 (PMC6066925; doi:10.1186/s13059-018-1483-4)
Supplement: Supplementary file 1 — Chromatin loop anchors are associated with genome instability in cancer and recombination hotspots in the germline. Figures S1–S8 and Tables S1–S4. (PDF 641 kb) [file 13059_2018_1483_MOESM1_ESM.pdf]

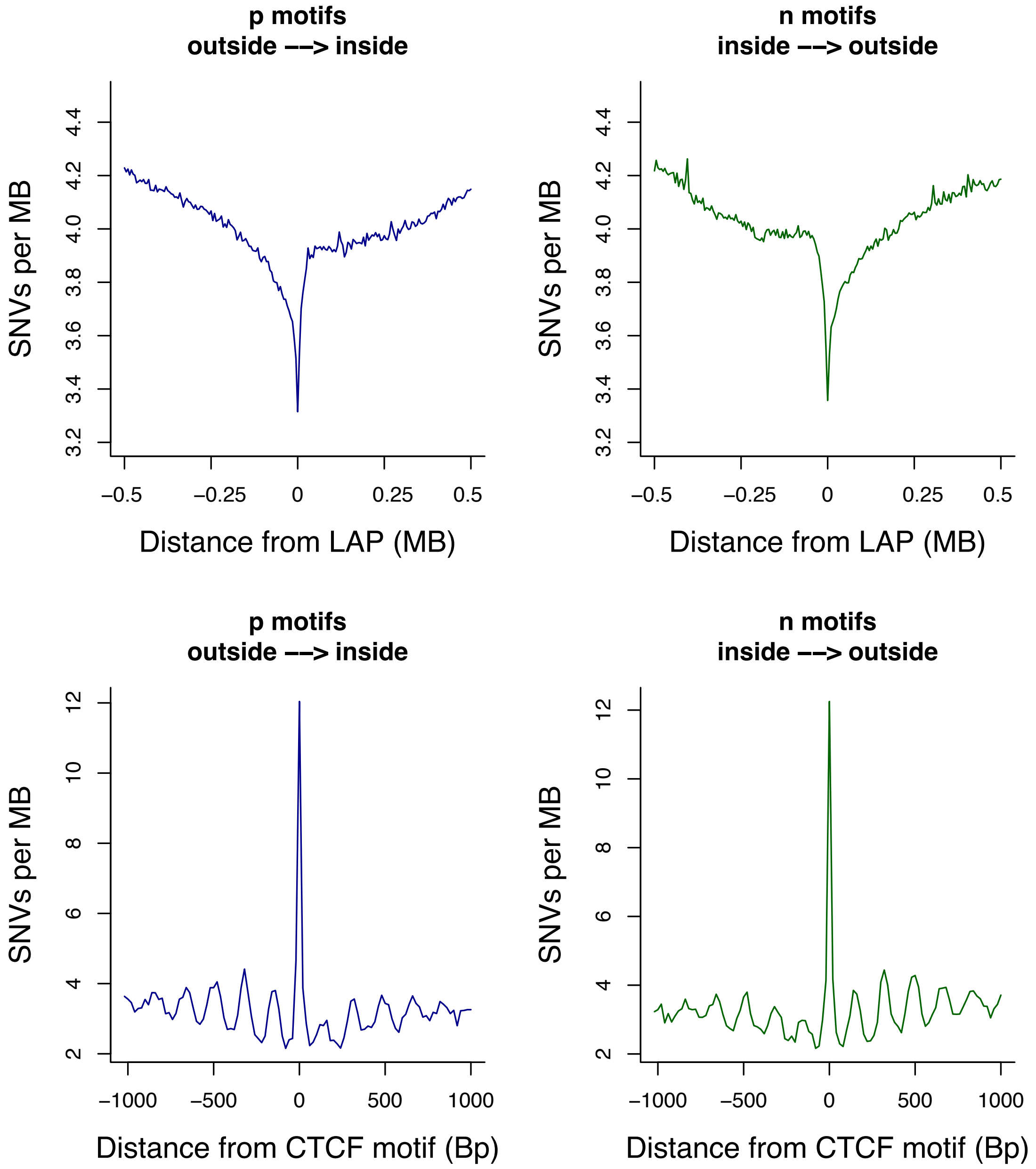

Figure S1: Average SNV rates per tumour, plotted separately for "p motifs", localised to the upstream loop anchor on the forward strand, *versus* "n motifs", localised to the downstream loop anchor on the reverse strand (Rao et al. 2014).

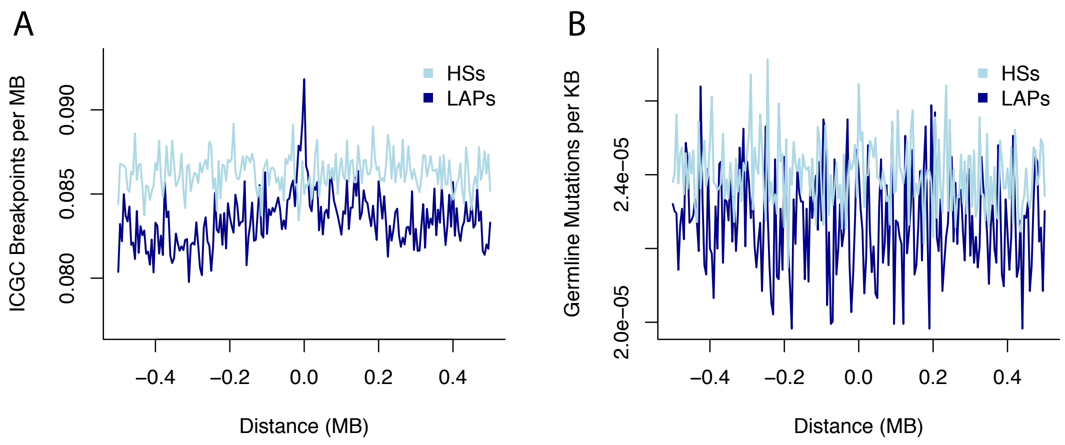

Figure S2: Germline mutation rates contrast with somatic mutation rates at LAPs. (A) DNA breakpoints in the ICGC pan-cancer analysis increase at LAPs but not at recombination HSs. (B) *De novo* germline variants from the DECODE trios dataset show no increase at LAPs or HSs. Light blue: genomic regions centred around recombination HSs; dark blue: centred around LAPs.

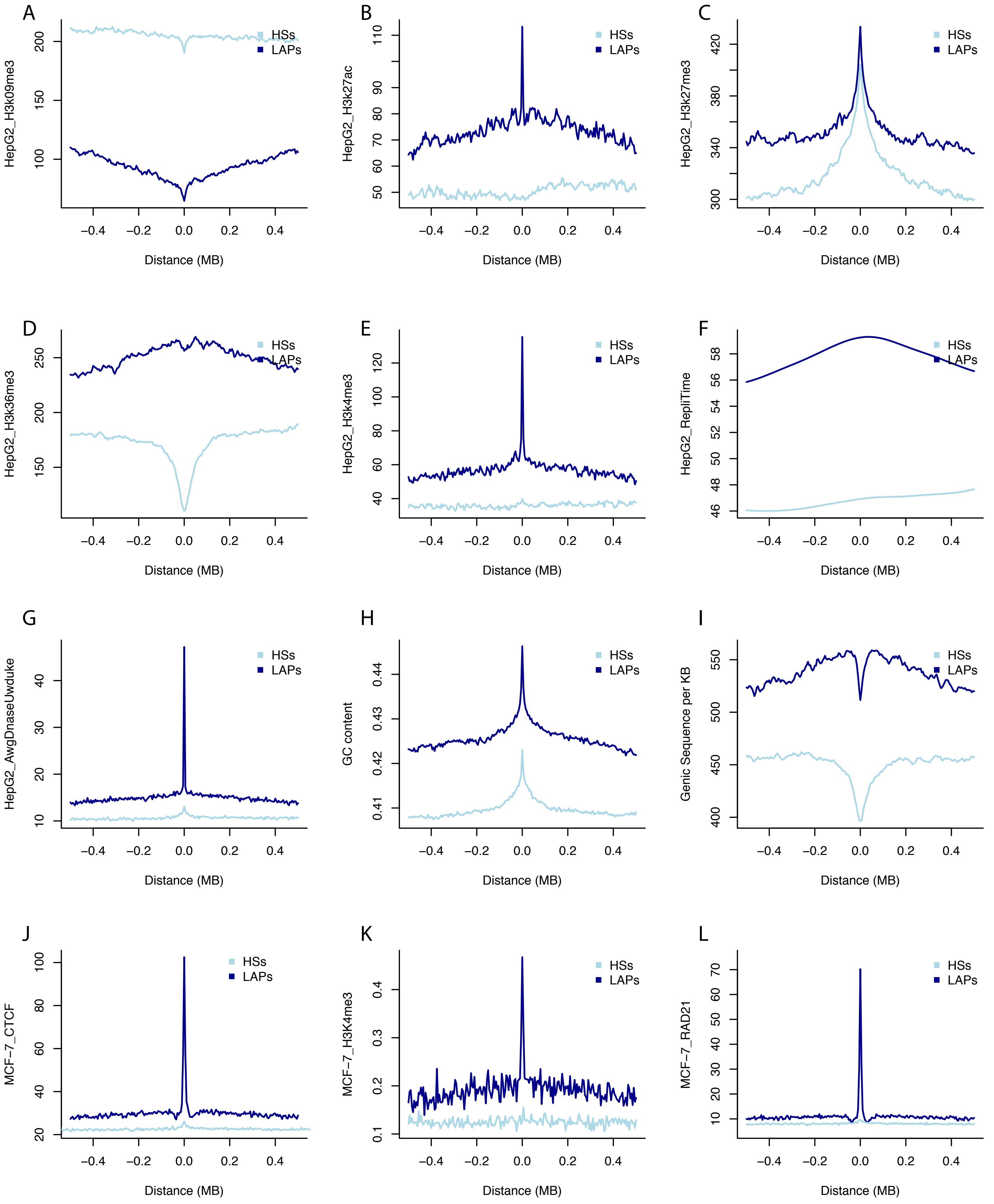
A
HepG2_H3k09me3
100
150
200
HSs
LAPs
−0.4
−0.2
0.0
0.2
0.4
Distance (MB)
B
HepG2_H3k27ac
50
60
70
80
90
110
HSs
LAPs
−0.4
−0.2
0.0
0.2
0.4
Distance (MB)
C
HepG2_H3k27me3
300
340
380
420
HSs
LAPs
−0.4
−0.2
0.0
0.2
0.4
Distance (MB)
D
HepG2_H3k36me3
150
200
250
HSs
LAPs
−0.4
−0.2
0.0
0.2
0.4
Distance (MB)
E
HepG2_H3k4me3
40
60
80
100
120
HSs
LAPs
−0.4
−0.2
0.0
0.2
0.4
Distance (MB)
F
HepG2_RepliTime
46
48
50
52
54
56
58
HSs
LAPs
−0.4
−0.2
0.0
0.2
0.4
Distance (MB)
G
HepG2_AwgDnaseUwduke
10
20
30
40
HSs
LAPs
−0.4
−0.2
0.0
0.2
0.4
Distance (MB)
H
GC content
0.41
0.42
0.43
0.44
HSs
LAPs
−0.4
−0.2
0.0
0.2
0.4
Distance (MB)
I
Genic Sequence per KB
400
450
500
550
HSs
LAPs
−0.4
−0.2
0.0
0.2
0.4
Distance (MB)
J
MCF−7_CTCF
20
40
60
80
100
HSs
LAPs
−0.4
−0.2
0.0
0.2
0.4
Distance (MB)
K
MCF−7_H3K4me3
0.1
0.2
0.3
0.4
HSs
LAPs
−0.4
−0.2
0.0
0.2
0.4
Distance (MB)
L
MCF−7_RAD21
10
20
30
40
50
60
70
HSs
LAPs
−0.4
−0.2
0.0
0.2
0.4
Distance (MB)

Figure S3: Variation in chromatin features around LAPs and recombination HSs. Average overlap, per Kb, with HepG2 cell line ChIP-seq peaks around LAPs and HSs for (A) H3K9me3, (B) H3K27ac, (C) H3K27me3, (D) H3K36me3, (E) H3K4me3. (F) Average replication timing in HepG2. (G) Average overlap per Kb with HepG2 DNAse HS sites; (H) Average GC content; (I) Average genic sequence per Kb. Average overlap, per Kb, with MCF-7 cell line ChIP- seq peaks around HMEC LAPs and HSs for (J) CTCF, (K) H3K4me3, (L) RAD21. Light blue: centred around recombination HSs; dark blue: centred around LAPs.

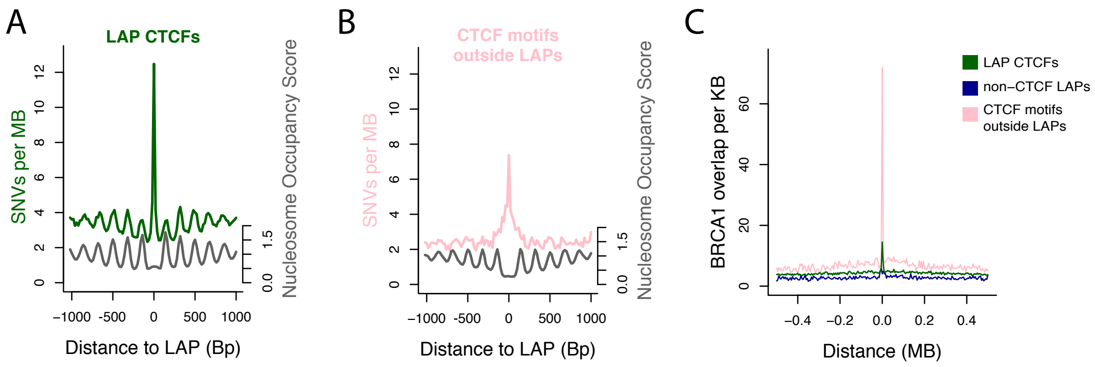

Figure S4: A comparison of mutational patterns and BRCA1 binding around different classes of CTCF sites and LAPs, i.e. CTCF sites within LAPs (A, C); CTCF sites outside LAPs (B, C); LAPs that do not contain CTCF motifs or evidence for CTCF binding in ENCODE ChIP-seq data across 18 cell types (Zerbino et al. 2015) (C). A, B) ICGC SNV rates per individual; C) BRCA1 ChIP-seq binding overlap. In panel C, BRCA1 binding is plotted around tissue-matched HMEC LAPs (green line), whereas the union set of LAPs was used in panels A and B.

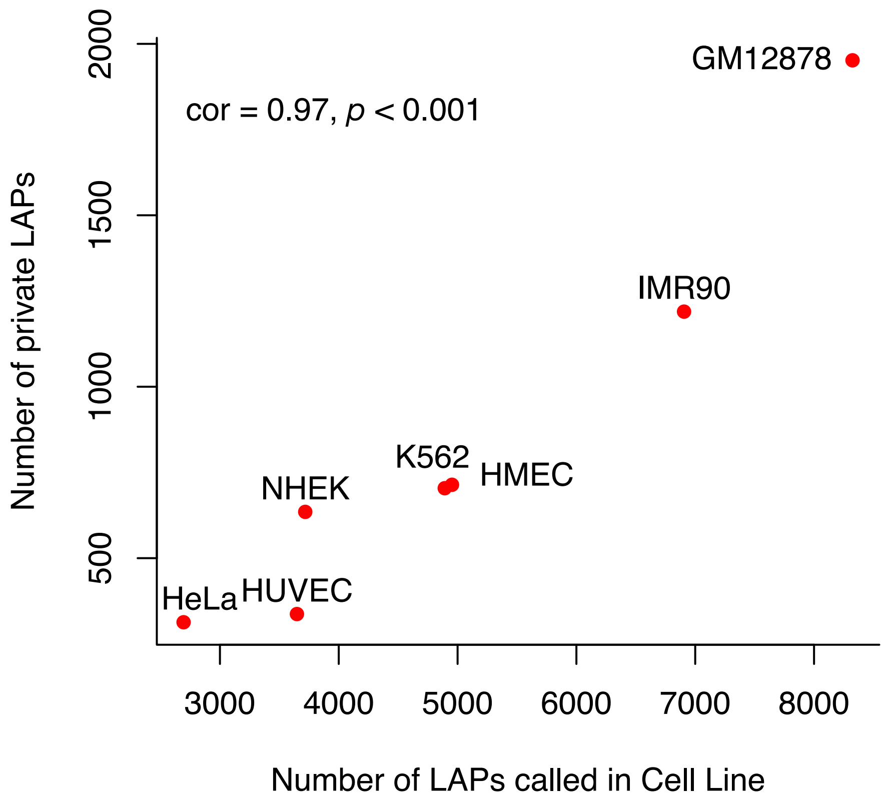

Figure S5: LAP discovery is a function of Hi-C sequencing depth.

The number of private, i.e. cell line-specific, CTCF binding site containing LAPs plotted against the total number of LAPs called in each cell line assayed by Rao et al. (2014).

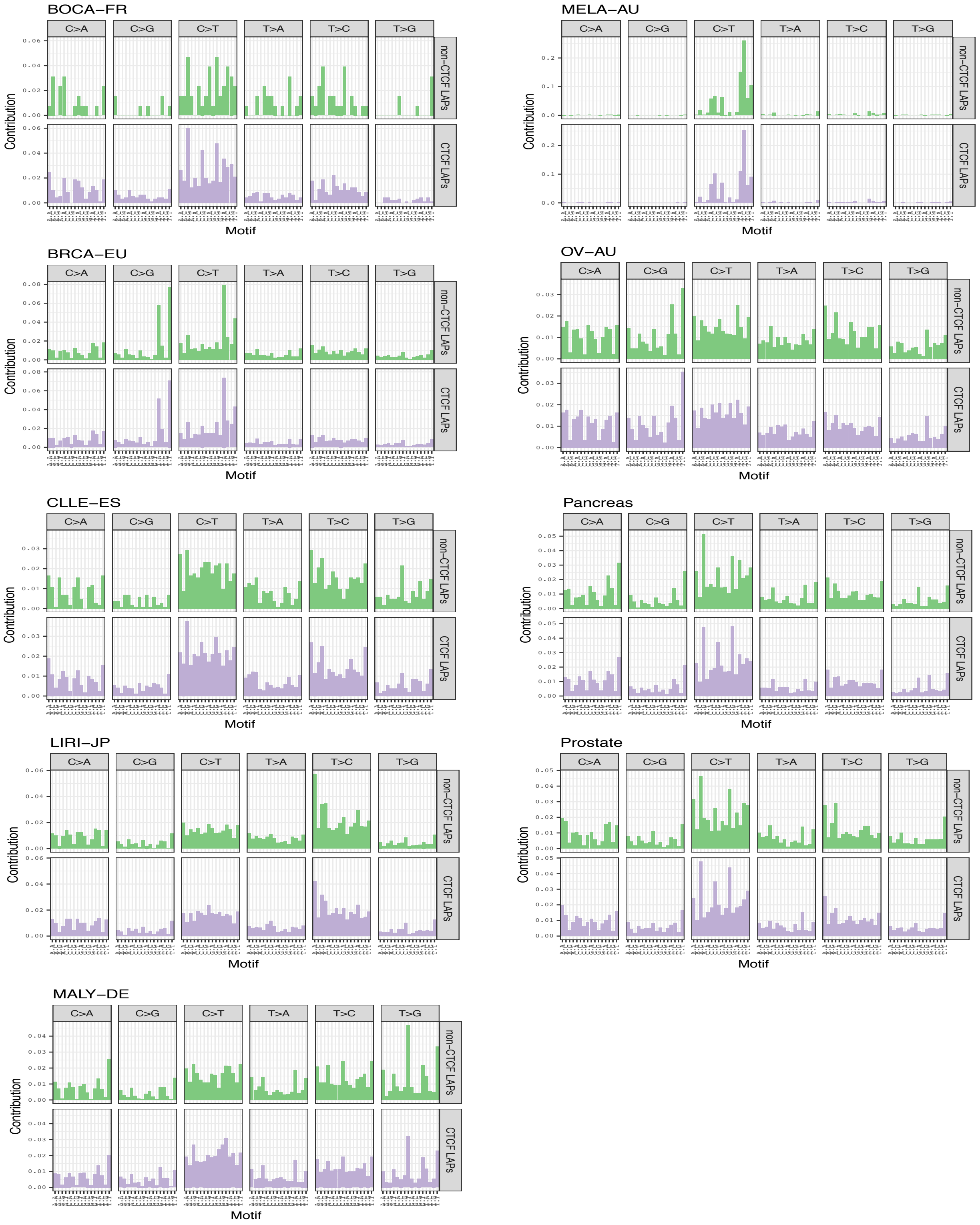

Figure S6: Mutational Spectra at non-CTCF LAPs (top rows) and CTCF-LAPs (bottom rows). Shown are the rates of somatic mutation at each of the 96 trinucleotide contexts, for each tissue type separately.

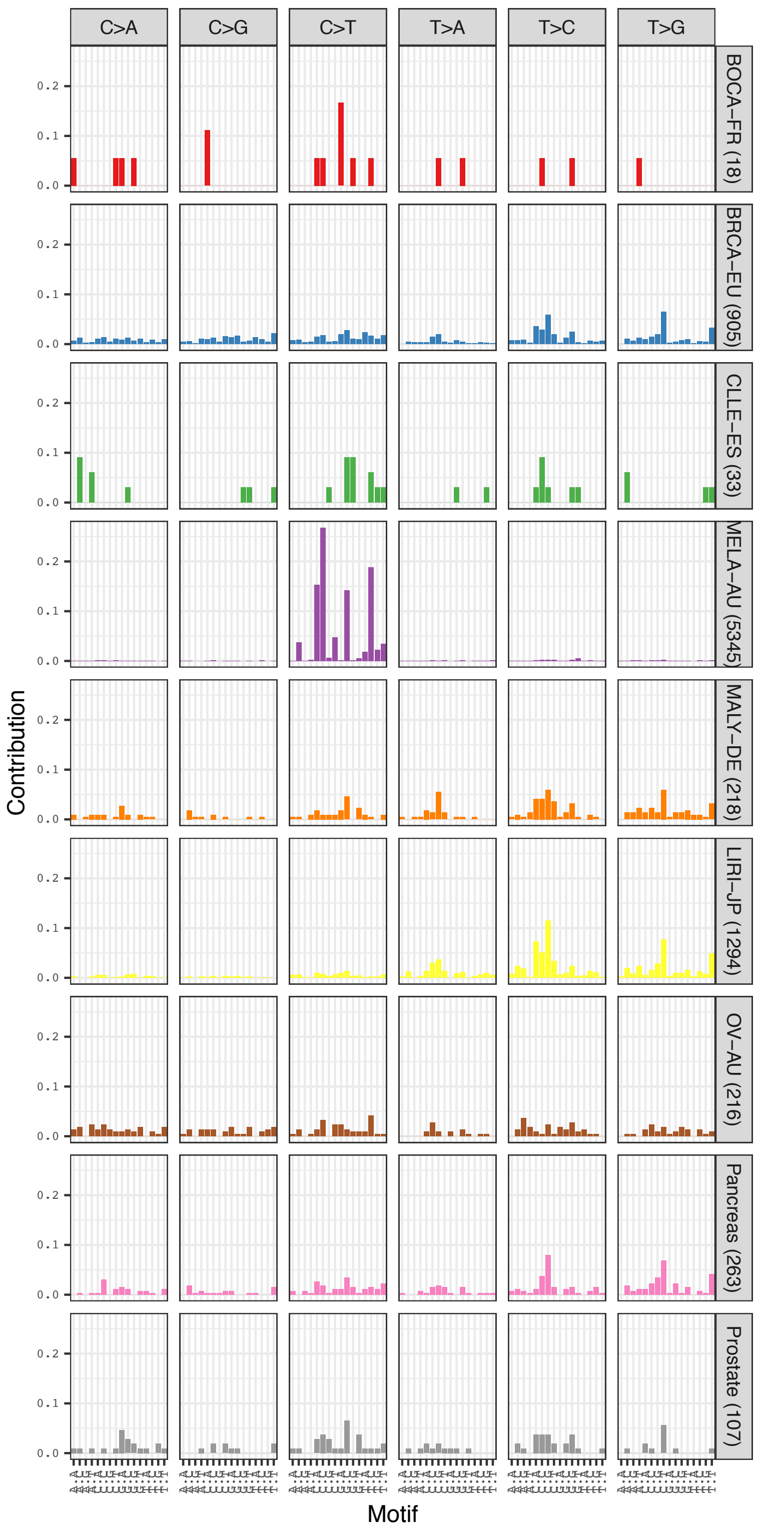

Figure S7: Mutational Spectra at CTCF motifs inside the union set of LAPs. Shown are the rates of somatic mutation at each of the 96 trinucleotide contexts. The number in brackets indicates the total number of somatic mutations found at CTCF sites in the respective tissue type.

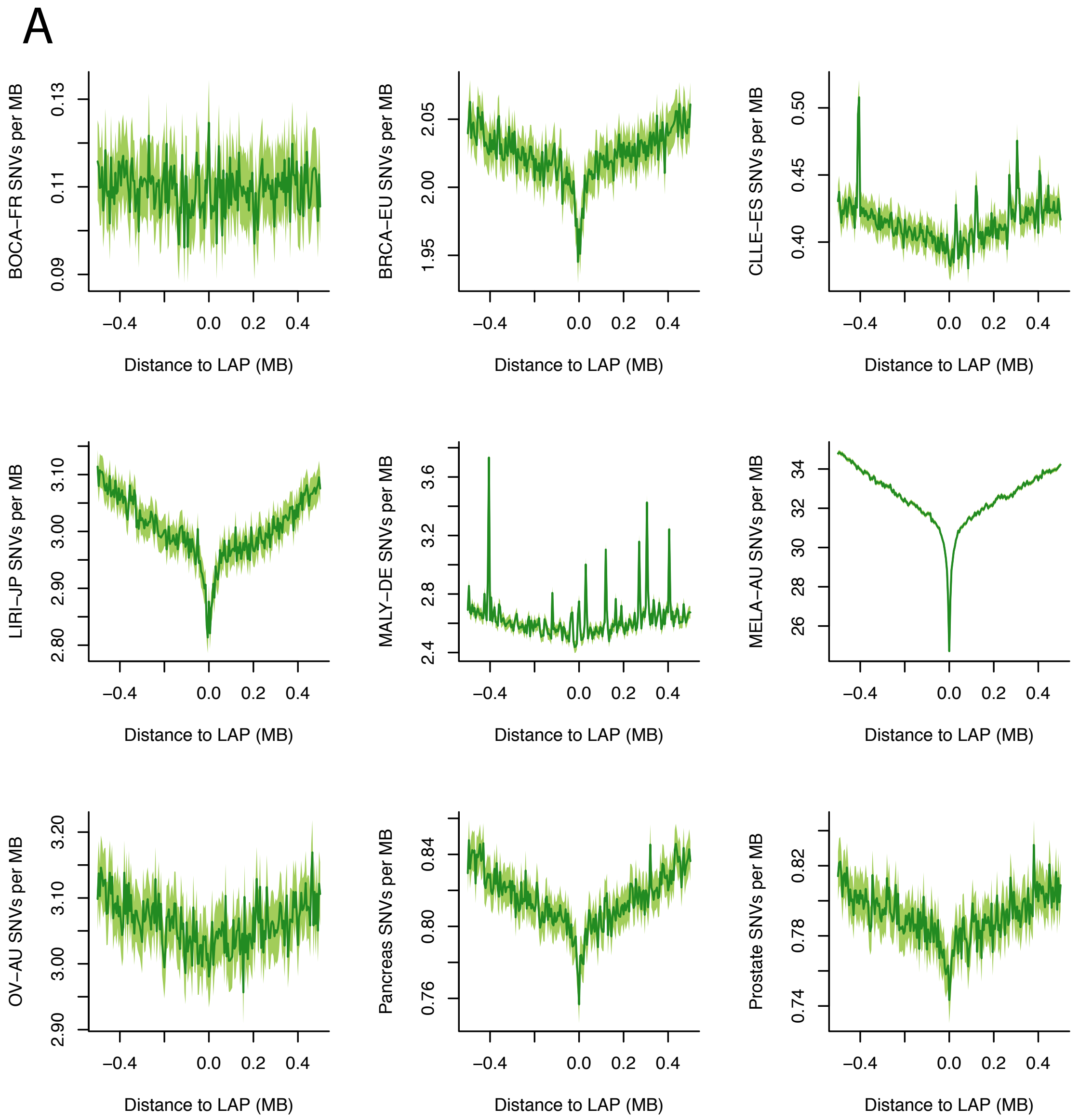
A
BOCA–FR SNVs per MB
0.09
0.11
0.13
BRCA–EU SNVs per MB
1.95
2.00
2.05
CLLE–ES SNVs per MB
0.40
0.45
0.50
LIRI–JP SNVs per MB
2.80
2.90
3.00
3.10
MALY–DE SNVs per MB
2.4
2.8
3.2
3.6
MELA–AU SNVs per MB
26
28
30
32
34
OV–AU SNVs per MB
2.90
3.00
3.10
3.20
Pancreas SNVs per MB
0.76
0.80
0.84
Prostate SNVs per MB
0.74
0.78
0.82
−0.4
0.0
0.2
0.4
Distance to LAP (MB)

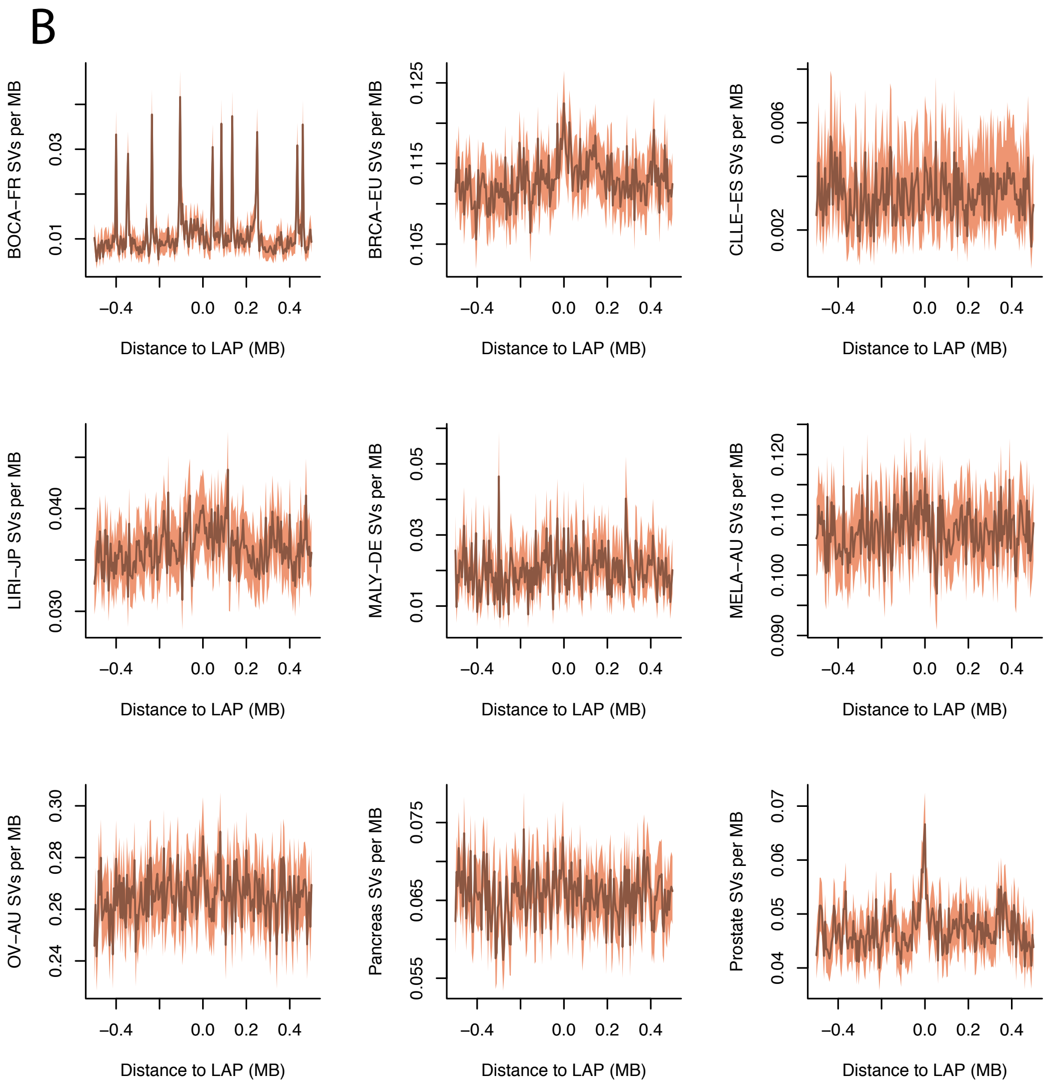

Figure S8: Mutational landscapes vary among tumour types. (A) SNV rates at LAPs, stratified by cancer type. (B) SV Breakpoint rates at LAPs, stratified by cancer type.

Table S1: Random Forest regression of the outcome variable "BRCA1 binding" in MCF-10A cells and "RAD51 binding" in MCF-7 cells near HMEC LAPs. Shown is the % increase in mean square error (%IncMSE) when each input variable is randomly permuted.

BRCA1 binding		RAD51 binding	
**Input Variable**	**%IncMSE**	**Input Variable**	**%IncMSE**
X1_Active_Promoter	45.81	X13_Heterochrom	18.08
X2_Weak_Promoter	21.68	X5_Strong_Enhancer	15.32
X15_Repetitive	19.84	X11_Weak_Txn	14.37
X13_Heterochrom	15.39	X2_Weak_Promoter	12.92
X14_Repetitive	15.23	X10_Txn_Elongation	10.53
X4_Strong_Enhancer	14.98	X12_Repressed	10.25
X10_Txn_Elongation	13.11	X8_Insulator	9.97
X12_Repressed	12.16	X7_Weak_Enhancer	9.31
X8_Insulator	11.96	X1_Active_Promoter	8.90
X5_Strong_Enhancer	11.90	X6_Weak_Enhancer	8.20
X7_Weak_Enhancer	11.80	X4_Strong_Enhancer	8.11
X6_Weak_Enhancer	8.55	X9_Txn_Transition	7.68
X9_Txn_Transition	7.74	X3_Poised_Promoter	4.09
X11_Weak_Txn	7.48	X15_Repetitive	3.76
X3_Poised_Promoter	3.44	X14_Repetitive	3.15
LAP_overlap	-13.81	LAP_overlap	-11.21

Table S2: The SNV and SV dataset. Shown are the number of icgc tumours used in this study, and the total numbers of somatic mutations in these samples.

	# SNV Donors	# SNVs	# Structural donors	# Structural Variants
BOCA-FR	98	36,374	97	2,470
BRCA-EU	560	3,479,652	544	155,300
CLLE-ES	218	348,176	92	756
EOPC-DE	62	87,912	11	686
LIRI-JP	258	2,691,085	243	22,324
MALY-DE	100	1,026,653	26	1,574
MELA-AU	183	21,442,747	183	57,350
OV-AU	93	913,650	93	64,126
PACA-AU	391	1,126,163	159	40,664
PAEN-AU	52	139,278	51	2,384
PAEN-IT	37	106,592	37	2,790
PRAD-CA	124	306,740	123	7,718
PRAD-UK	108	400,786	13	10,338
SUM	2,284	32,105,808	1,672	368,480

Table S3: Random Forest modelling of the outcome variable "mutation rate" per 5kb window, within 1MB of the union set of LAPs. Input variables for each cancer type are: local GC content, 1KG recombination rate, average replication timing across 15 tissue types, CTCF motif occurrence, LAP presence and quadruplex sequence overlap. The % increase in mean square error of the model (%IncMSE) is shown when each variable is randomly permuted. a) SNV rates; b) SV rates.

a) SNVs

**%IncMSE**	**BOCA-FR**	**BRCA-EU**	**CLLE-ES**	**LIRI-JP**	**MALY-DE**	**MELA-AU**	**OV-AU**	**Pancreas**	**Prostate**
GC	20.19	41.95	18.39	44.40	7.34	50.75	19.15	32.44	23.67
RecRate	3.07	19.26	8.42	31.53	5.31	52.29	19.48	22.59	14.20
RepliTime	24.77	131.70	76.86	229.27	45.19	169.73	126.42	166.67	79.10
CTCF	7.72	-0.80	2.18	9.49	1.99	21.53	6.35	9.19	6.97
LAPs	1.41	7.62	0.46	16.12	0.32	57.25	8.65	15.55	9.00
Quadruplex	25.04	24.96	20.25	32.99	4.73	32.27	25.34	19.40	23.48

b) SVs

**%IncMSE**	**BOCA-FR**	**BRCA-EU**	**CLLE-ES**	**LIRI-JP**	**MALY-DE**	**MELA-AU**	**OV-AU**	**Pancreas**	**Prostate**
GC	9.87	22.31	8.60	12.88	9.41	20.24	22.88	20.13	11.87
RecRate	3.80	9.38	1.29	3.04	-0.06	4.13	2.95	10.60	6.61
RepliTime	5.39	16.40	4.04	12.54	6.09	17.29	4.73	19.61	10.03
CTCF	-0.27	8.51	-3.57	-2.66	2.38	2.00	5.39	2.50	7.09
LAPs	-2.20	7.47	-1.33	2.12	-0.79	3.53	1.80	3.00	4.52
Quadruplex	10.44	20.57	5.78	9.82	10.70	16.60	18.23	21.13	8.98

Table S4: Functional annotation enrichments at recurrently disrupted LAPs. Enrichment of genes within the foreground set of 398 genomic regions (chromatin loops recurrently affected by structural variant breakpoints), relative to the background set (9,973 loops defined by all 14,737 LAPs) for GO and pathway terms. All regions were associated with genes using the GREAT tool (see Methods).

Term	FDR Q-Val	Fold Enrichment	Foreground Region Hits	Total Regions	Foreground Gene Hits	Total Genes Annotated
**GO Biological Process**						
negative regulation of protein kinase activity	5.99E-02	2.64	26	247	21	174
negative regulation of protein phosphorylation	5.42E-02	2.28	32	351	27	229
negative regulation of kinase activity	6.59E-02	2.47	26	264	21	184
positive regulation of mammary gland epithelial cell proliferation	5.50E-02	12.53	5	10	2	5
negative regulation of phosphate metabolic process	4.58E-02	2.06	37	450	32	293
negative regulation of transferase activity	4.75E-02	2.4	26	272	21	195
**GO Cellular Component**						

cyclin-dependent protein kinase holoenzyme complex	4.37E-02	6.07	8	33	6	17
**MSigDB Pathway**						
Influence of Ras and Rho proteins on G1 to S Transition	2.53E-04	8.08	10	31	7	26
Cdk2, 4, and 6 bind cyclin D in G1, while cdk2/cyclin E promotes the G1/S transition	1.39E-02	12.53	5	10	3	15
Chronic myeloid leukemia	4.59E-02	3.22	14	109	11	74
