# Supplementary material for: Chromatin loop anchors are associated with genome instability in cancer and recombination hotspots in the germline
Source: Genome Biol. 2018 Jul 30;19:101. doi: 10.1186/s13059-018-1483-4 (PMC6066925; doi:10.1186/s13059-018-1483-4)
Supplement: Supplementary file 2 — Review history of this manuscript. (DOCX 199 kb) [file 13059_2018_1483_MOESM2_ESM.docx]

**First round of review**

**Reviewer 1**

**Are the methods appropriate to the aims of the study, are they well described, and are necessary controls included?**

Generally yes, but more care should be taken to understand the correlations and respective contribution of the covariates to the signal observed,

**Are the conclusions adequately supported by the data shown?**

The use of early PCAWG data may be problematic. Not sure if a version of the dataset can be provided.

**Are sufficient details provided to allow replication and comparison with related analyses that may have been performed?**

The use of early PCAWG data may be problematic. Not sure if a version of the dataset can be provided.

**Does the work represent a significant advance over previously published studies?**

No

**Is the paper of broad interest to others in the field, or of outstanding interest to a broad audience of biologists?**

Yes: The genomic community is definitely interested in such analysis integrating a large variety of datasets and will appreciate the exhaustive list of references. the novelty is however more arguable.

General Comments

The report by Kaiser and Semple presents a comprehensive analysis of the mutational landscape around Chromatin Loop Anchor points (LAPs). They use publicly available datasets to define the LAPs (Rao et al) and calculate mutation rate from somatic SNV and Structural Variants in 2284 tumors from the ICGC datasets. They further compare and contrast the findings to other genomics regions such as recombination hotspots from the 1KG project, predicted CTCF binding motifs, known CTCF binding sites, known BRCA1 binding sites and location of double strand breaks. The report is likely a natural follow-up of their 2016 study (ref 21) which reported, using a similar dataset, that CTCF was the most affected motifs by somatic mutations. I found the paper well written and the analysis well conducted. I was more confused by the overall rationale for the study, the lack of significant novel findings in regards to ref 21 and ref 16, and the lack of consideration/insights of the potential strong correlation between the different features they analyzed. I present below more specific points that the authors would need to address.

Specific Comments

The authors present a very well documented study, citing many references to support their findings. But there is a strong overlap between LAP and other, more studied, genomics regions such as promoter, enhancers, HS, CTCF binding sites. As a consequence, this is really not clear which of the reported findings are truly novel, revealing some new functional role associated with LAPs, from which ones are merely confirmation of previous reports studying regions correlated with LAPs, including the previous study form the same authors (ref 21). Most CTCF binding sites are found in LAPs. And most LAPs are bound by CTCF. The authors need to present more clearly what are the novel findings specific to this report.

The authors present the ICGC dataset without much details, limiting the possible reproducibility of their analysis. Can they provide more information on the version of the dataset used? Was this dataset the result of the PCAWG consortium effort (Pan-Cancer Analysis of Whole Genome) or only individual ICGC projects. The PCAWG series of papers are currently likely under-review so the authors cannot provide a clear description of the data they used and how it was analyzed. While consortium like PCAWG may release early versions of the data, their final report contains precious information about quality control, black-listed samples, and specific empirical filtering of SNV and SV. Large genomic datasets have been reported to have specific artifacts that early or secondary users may overlook. Can the authors provide more details in their use of an early pre-published datasets? Did they perform some minimal quality control and filtering of the data?

Through figure 1 and 2, the authors present the main results of their analysis, which is the specific depletion of SNV in LAPs, enrichment of SNV in CTCF within LAPs and increased SV rate in LAPs. However, there is little contrast provided for this analysis, apart from the local rate around the region considered. Further down in the report, to address other questions, the authors used more appropriate methods. Specifically, the authors would need to present earlier, the contribution of factors known to impact mutational rates such as replication timing, GC content, promoter and enhancers, using some regression or machine learning method, or more empirically using permutation methods. In any case, the significance of the findings cannot be properly evaluated without controlling for these known sources of variation.

On the similar note, I believe the most rigorous analysis presented by the authors is the modeling performed on the breast dataset, where they used Random Forest to determine the contribution of multiple co-variates to the mutational rate of LAPs. While I understand that the same analysis cannot be performed on the Pan-Cancer results, lacking most chromatin marks, a similar analysis can be done, using GC content, replication timing, motifs, LAP overlap, CTCF binding, cancer type, cancer mutation rate. This would provide considerably more insight on the earlier section of the manuscript, where correlation between variables and cancer specific effects are a significant concern.

As determined by the authors in a few instances, the LAP dataset may have a number of false negative for their analysis, either from insufficient Hi-C coverage, or from the lack of Hi-C data in an appropriate tissue. This likely confound their comparison, such as non-CTCF-LAP vs CTCF-LAP presented in fig 6. The authors need to provide a measurable sense of how the LAP definition impacts their findings. The authors could for example present results on different sets of LAPs with different level of confidence, generated from union, sample specific, or intersection from down-sampled Hi-C data.

In the association with BRCA1/2 binding in breast cancer, as the authors points out, active transcription can explain the association. Similar to the previous comments above, the authors need to provide more insight by using a regression-based method to control for transcription rate, or from the 15 ChromHMM type of regions derived by ENCODE/HMEC. Is their finding at LAPs somewhat independent or is their observation only a correlation with known factors?

In figure 4B, the control by shifting by 50kb is interesting, and indeed likely corrects for replication timing and nuclear compartment. What is not clear however is the contribution of more local factors such as GC content, presence of PRDM9 or CTCF binding sites or motifs. Can the authors refine the observed association by looking at the contribution of known, tissue independent factors?

Since LAPs have a higher rate of DSB, do the SNV in the CTCF match a particular signature, such as the APOBED signature seen around Kataegis sites. Can the authors comment whether they believe the higher rate of SNV in CTCF is likely a consequence of cell autonomous processes such as APOBEC or DNA repair?

The comparison between the three categories of LAPs presented in Fig5A, is difficult to interpret as these 3 categories do not seem to be mutually exclusive. Wouldn't this analysis be more powerful and likely more interesting, if the observations were better contrasted in non-overlapping sets of LAPs?

The striking differences between Cancer types presenting in Figure S7 is concerning. While a Pan-Cancer dataset increases statistical power, its analysis can be confounded when strong differences exist between the cohort sizes and mutational patterns as suggested in Table S1. The authors need to discuss how the differences may impact their observations and ideally take cancer type into consideration using a regression model.

In Fig 6B, the authors claim the high fragility at non-LAP CTCF, but the data presented seems particularly noisy. Can the authors present a statistical evaluation to determine if the enrichment is significant?

Minor Points

Despite a very comprehensive list of referenes, the autors missed important early reports documenting, likely for the first time, the association between copy number breakpoint and chromatin loops from Hi-C. ( https://www.ncbi.nlm.nih.gov/pmc/articles/PMC3460994/ and https://www.ncbi.nlm.nih.gov/pubmed/22101486). These references (and ref 16) may diminish some of the novelty of their findings regarding DSB enrichment at LAPs and their work should be discussed with these in perspective.

In the title, and abstract (line 33) the authors state the association between LAPs and HS is high and unexpected, but the overlap is only 16%. Can the authors clarify whether the LAPs are significantly associated with HS and if not the case, edit the title and abstract?

Line 61: Cohesion should be cohesin (autocorrect error)

The abbreviation of kilobase is kb no Kb.

In Table1 why is the expected only for the triple and quadruple intersection? Can the authors provide numbers for the other comparisons? Can the authors provide the results of the statistical analysis either using hypergeometric test or the FDR permutation-based analysis?

In contrast to their claim online 270, some of the Hi-C data should actually be fairly relevant. For example, GM12878 is a lymphoblastic cell line and may relate well to the CLL dataset. Similarly, NHEK may relate to Melanoma. Can the authors discuss more thoroughly why they thought HUVEC/Breast was a better histology to focus on?

The details of the Gene-Set enrichment analysis should be presented in the method section (they are only briefly mentioned in the supplemental table header). The selection of the control dataset for enrichment as well as the gene set tested should be more explicit.

Line 317: clarify what recurrently disrupted mean in this context. Number of breast cancer donors?

The method section needs to be more structured, so that the sub-sections related to specific analysis can be easily identified.

**Reviewer 2**

**Are the methods appropriate to the aims of the study, are they well described, and are necessary controls included?**

Yes

**Are the conclusions adequately supported by the data shown?**

Yes

**Are sufficient details provided to allow replication and comparison with related analyses that may have been performed?**

Yes

**Does the work represent a significant advance over previously published studies?**

Yes

**Is the paper of broad interest to others in the field, or of outstanding interest to a broad audience of biologists?**

Yes: Breakpoints in cancer are of central interest.

This is an interesting study of the association of LAPs with SNVs, SVs, chromatin marks, CTCF binding sites, and meiotic recombination hotspots in cancer versus germline cells. The conclusions are supported by the data. I have a few comments which would make the paper stronger.

Major:

1. Of the overlapping 16% LAPs and HSs, what percentage have PRDM9 core motifs?

2. Are LAPs increased at cancer fragile gene sites (e.g. WWOX, FHIT, A2BP1)?

3. Are non-CTCF LAPs enriched for other binding motifs?

4. The Spearman values in Fig. 7C are extremely low, it's not clear whether these are meaningful.

5. It's not clear exactly how LAPs were defined/identified.

Minor:

1. Fig 1B missing labels of the different lines.

2. Need to properly introduce the 1KG dataset before describing its results.

**Responses to Reviewer #1**

*Reviewer #1: General Comments

1. The report by Kaiser and Semple presents a comprehensive analysis of the mutational landscape around Chromatin Loop Anchor points (LAPs). They use publicly available datasets to define the LAPs (Rao et al) and calculate mutation rate from somatic SNV and Structural Variants in 2284 tumors from the ICGC datasets. They further compare and contrast the findings to other genomics regions such as recombination hotspots from the 1KG project, predicted CTCF binding motifs, known CTCF binding sites, known BRCA1 binding sites and location of double strand breaks. The report is likely a natural follow-up of their 2016 study (ref 21) which reported, using a similar dataset, that CTCF was the most affected motifs by somatic mutations. I found the paper well written and the analysis well conducted. I was more confused by the overall rationale for the study, the lack of significant novel findings in regards to ref 21 and ref 16, and the lack of consideration/insights of the potential
strong correlation between the different features they analyzed. I present below more specific points that the authors would need to address.* 

**Response:**

We thank the reviewer for their positive remarks about our analysis and writing, but we believe that both the rationale for our study and the vast majority of our findings are novel. To our knowledge there has been no previous study of the somatic mutational landscape at chromatin loop anchor points (LAPs) genome-wide, based upon the large whole genome sequencing (WGS) datasets for tumours accumulated by ICGC. Similarly, there has been no study of the patterns of recombination at LAPs. Studying the patterns of somatic and germline variation associated with LAPs was therefore the rationale for this study.

Our novel findings are as follows:

- LAP regions are strongly depleted for single nucleotide variants (SNVs) in tumours (largely as a function of replication timing), in stark contrast to the precise local elevations seen at CTCF sites within these regions (as in ref 21). These opposing patterns at the same regions have not been reported before.
- In spite of low SNV rates, LAP regions are notably enriched for tumour structural variant (SV) breakpoints, as seen in separately sequenced cancer patient cohorts. This is consistent with the patterns of *in vitro* double strand break data produced by the experimental study of LAPs that we reference (ref 16), but our analysis is completely independent of the analysis in ref 16, and is the first genome-wide study of tumour breakpoints at LAPs.
- Having established that LAPs are prone to tumour breakpoints, we show that recurrently disrupted LAPs in tumours are often associated with genes annotated with functions in cell cycle transitions. This observation is a novel contribution to the debate about disrupted chromatin domains in cancers, and suggests that hypotheses involving selection are not necessary to explain the disruptions seen.
- A substantial fraction of LAPs (16%) overlap known meiotic recombination hotspot (HSs) precisely and are enriched for the core PRDM9 binding motif. This area has not been explored before, and the association of recombination hotspots with a class of LAPs is entirely novel.

*2. The authors present a very well documented study, citing many references to support their findings. But there is a strong overlap between LAP and other, more studied, genomics regions such as promoter, enhancers, HS, CTCF binding sites. As a consequence, this is really not clear which of the reported findings are truly novel, revealing some new functional role associated with LAPs, from which ones are merely confirmation of previous reports studying regions correlated with LAPs, including the previous study form the same authors (ref 21). Most CTCF binding sites are found in LAPs. And most LAPs are bound by CTCF. The authors need to present more clearly what are the novel findings specific to this report.*

**Response:**

We have addressed the issue of novelty above. In this manuscript, we investigate mutational patterns around LAPs, which are genomic structures of several kb in size, and, as the reviewer points out, these regions overlap many other genomic features of interest in the genome. In common with many others, we have used random forest regression modelling to disentangle the varying influences of these features. The pattern of SNVs at LAPs that we observe in the current manuscript is depletion over a broad region (Fig. 1a), super-imposed upon the more local elevations at CTCF sites seen previously (Fig. 1b; ref 21). This opposing pattern of tumour SNV rates at LAPs and the CTCF sites embedded within LAPs is novel, and the depletion at LAPs cannot reasonably be caused by increased rates at CTCF sites within those LAPs. In addition, we show that DNA breakage in tumours is not dependent on the presence of CTCF (Fig. 6b) and that double strand breakage does not depend on the presence of promoters or enhancers at LAPs (Fig. 3), but it is seen across all loop anchor points. In particular, we show that LAPs lacking CTCF motifs and lacking any CTCF ChIP-seq signal still suffer higher rates of SV breakpoints (Fig. 6). We also show that the presence of other regions expected to be prone to mutation at LAPs, such as regions carrying histone modifications (H3K4me3, H3K27ac) associated with active promoters and enhancers, affects mutation rates as expected (Fig. 7). However, these models also show that the presence of enhancers and promoters are not the main influences on the rates of mutation seen at LAPs (Fig. 7).

As mentioned above, the high rates of somatic SV breakpoints at LAPs in tumour WGS datasets is also a novel observation, though it is broadly consistent with *in vitro* experimental data on double strand break rates in mouse and human cell cultures (ref 16). We consider this consistency with independent experimental data to be encouraging, and do not believe it compromises the novelty of our observations. Note that Canela et al (ref 16) did not consider the genome-wide relationship between tumour SV breakpoints and LAPs. Instead, they discussed the agreement between seven, well studied tumour translocation breakpoint regions (see Canela et al, Fig1 and FigS3) and the DSB data they generated; they also noted elevated rates of experimentally induced DSBs at LAPs (Canela et al, Fig4). Similarly, Kaiser et al (ref 16) also failed to examine the tumour mutational landscape at LAPs and instead focused on the mutational loads at a variety of binding sites. Neither ref 16 or ref 21 considered recombination patterns in any context.

Overall, we consider it not only novel but quite remarkable that LAPs emerge as foci for evolutionary change in tumours and in the recent evolution of human populations. We have attempted to make the novelty of our findings more obvious in the abstract of the revised manuscript.

*3. The authors present the ICGC dataset without much details, limiting the possible reproducibility of their analysis. Can they provide more information on the version of the dataset used? Was this dataset the result of the PCAWG consortium effort (Pan-Cancer Analysis of Whole Genome) or only individual ICGC projects. The PCAWG series of papers are currently likely under-review so the authors cannot provide a clear description of the data they used and how it was analyzed. While consortium like PCAWG may release early versions of the data, their final report contains precious information about quality control, black-listed samples, and specific empirical filtering of SNV and SV. Large genomic datasets have been reported to have specific artifacts that early or secondary users may overlook. Can the authors provide more details in their use of an early pre-published datasets? Did they perform some minimal quality control and filtering of the data?*

**Response:**

We apologise that the origin of the variant calls was not clearer. The dataset used was not the PCAWG consortium data, which is currently unpublished, instead we used the official ICGC data release 23 to achieve the largest, already published somatic mutation sample sizes available in April 2017. We have modified the Methods section to make this clearer: “Release_23 ICGC (https://dcc.icgc.org/) datasets with whole genome sequence derived calls for both SNV and SVs (not under publication moratorium in April 2017) were included.”

This dataset is an large collection of variant calls reviewed and published in a series of high impact ICGC publications, and since used in many other manuscripts, but we take the proposed issue of artefacts seriously. Although neither we nor the ICGC would claim that these variant call datasets are faultless, our results describe genome-wide trends in overall patterns of variants, and these trends are unlikely to be explained by artefacts. For instance, the patterns of SNV and SV rates per Mb we report (presented in Fig. 1a and Fig. 1c) involve significant shifts in mutation rates genome-wide, at 14,737 particular points independently annotated as LAPs based upon Hi-C data. Patterns of plausible artefacts would be expected to affect a small minority of variant calls, but, to affect our results, would have to afflict substantial proportions of SNV and SV calls across multiple studies. These artefacts would also not be expected to be enriched at regions independently annotated as LAPs, and would not be expected to be seen across the multiple patient cohorts included in this ICGC data release. Finally, analogous though often weaker, genome-wide influences of chromatin features have been reported previously, based upon collections of mutations orders of magnitude smaller, and have been subsequently replicated multiple times (e.g. Schuster-Böckler and Lehner, 2013).

*4. Through figure 1 and 2, the authors present the main results of their analysis, which is the specific depletion of SNV in LAPs, enrichment of SNV in CTCF within LAPs and increased SV rate in LAPs. However, there is little contrast provided for this analysis, apart from the local rate around the region considered. Further down in the report, to address other questions, the authors used more appropriate methods. Specifically, the authors would need to present earlier, the contribution of factors known to impact mutational rates such as replication timing, GC content, promoter and enhancers, using some regression or machine learning method, or more empirically using permutation methods. In any case, the significance of the findings cannot be properly evaluated without controlling for these known sources of variation.*

**Response:**

We structured the manuscript logically, such that we first establish the dominant patterns of mutations at LAPs (Fig. 1, Fig. 2) and then explore the underlying features influencing these patterns (subsequent figures). Also, in our opinion the best situation for comprehensive modelling of underlying features is a mutation dataset for a tumour type with matched chromatin and other data available for that tissue. This is why we focus on modelling breast tumour mutation rates in terms of features assayed in epithelial breast cells (Fig. 7). With the larger pan-cancer mutation dataset it is not possible to match tissue specific features to tumour type in the same way.

However, as requested by the reviewer, we have now added a random forest regression analysis of the pan-cancer dataset, including cell type-invariant features only (GC content, recombination rate, average replication timing, constitutive CTCF binding site overlap, union LAP presence and quadruplex sequence overlap). The results of this analysis are presented in the new Table S3, which shows the relative influences of the same cell type-invariant features on each tumour type. We note that the importance of “LAP presence” on mutation rate is consistent with the mutational profiles of the different tumour types near LAPs (Figure S8). We have also added this accompanying text to the results section:

“Cell-type specific features are not available for most of the cancer types studied, but we carried out random forest regression analysis on mutation rates in each of the nine tumour types separately, using, as input features, only cell type-invariant features around the union set of 14,737 LAPs (Additional File 2: Table S3). As expected, the previously reported associations between mutation rates and replication timing, GC content and quadruplex sequence are seen across all tumour types. The estimates of the influence of overlapping LAPs (the variable “LAPs” in Additional File 2: Table S3) on mutation rates directly reflects the dip - or lack thereof - of mutation rates at LAPs seen in a given tumour type (Additional File 1: Fig. S8). For example, in the case of melanoma, where a strong decline in SNV rates is evident, this variable influences mutation rates strongly as reflected in a high %MSE score. In contrast, in malignant lymphoma, where the SNV decline at LAPs is very modest, LAP overlap has little measurable effect on SNV rates.”

The following additional text was added to the Methods section: “Average Replication time was calculated from the Wavelet-smoothed Signals of the 15 Encode cell types available at the UCSC Genome Browser site (http://genome.ucsc.edu/).”

*5. On the similar note, I believe the most rigorous analysis presented by the authors is the modeling performed on the breast dataset, where they used Random Forest to determine the contribution of multiple co-variates to the mutational rate of LAPs. While I understand that the same analysis cannot be performed on the Pan-Cancer results, lacking most chromatin marks, a similar analysis can be done, using GC content, replication timing, motifs, LAP overlap, CTCF binding, cancer type, cancer mutation rate. This would provide considerably more insight on the earlier section of the manuscript, where correlation between variables and cancer specific effects are a significant concern.* 

**Response:**

As the reviewer points out, the tissue matched input variables used in the random forest analysis of the breast cancer dataset should not be used for the pan-cancer dataset, which is why we chose a more in-depth analysis on the breast cancer data for which epigenetic information is available. Below we show a table of results from analysis that includes “cancer type” as a covariate in a regression model, along with cell type-invariant features, to predict pan-cancer mutation rates. As expected, the importance of each “cancer type” was directly proportional to the number of mutations that each cancer type contributed to the pan-cancer mutation rate. Since these results mainly reflect the different sample sizes of mutations between tumour types, we have chosen to include the pan-cancer analysis in Table S3 (see response to point 4 above) in the manuscript, rather than the table below.

| Table: Random Forest Regression, modelling the outcome variables "pan-cancer SNV rates" and "pan-cancer SV rates", respectively. Input variables include the number of mutations contributed by each cancer type. | | | | | | | |  | |
| --- | --- | --- | --- | --- | --- | --- | --- | --- | --- |
| **Outcome variable: SNVs** |  |  | **Outcome variable: SVs** | |  | |  | |  |
| **Input Variable** | **%IncMSE** | **SNVs in cancer type** |  | **Input Variable** | | **%IncMSE** | | **SVs in cancer type** | |
| MELA.AU | 269.82 | 21,442,747 |  | SVs.OV.AU | | 352.27 | | 64,126 | |
| BRCA.EU | 66.58 | 3,479,652 |  | SVs.BRCA.EU | | 231.68 | | 155,300 | |
| LIRI.JP | 61.69 | 2,691,085 |  | SVs.Prostate | | 218.61 | | 18,742 | |
| MALY.DE | 40.43 | 1,026,653 |  | SVs.MELA.AU | | 171.8 | | 57,350 | |
| Pancreas | 35.17 | 1,372,033 |  | SVs.Pancreas | | 168.93 | | 45,838 | |
| Prostate | 30.69 | 795,438 |  | SVs.LIRI.JP | | 154.34 | | 22,324 | |
| OV.AU | 26.8 | 913,650 |  | SVs.BOCA.FR | | 66.52 | | 2,470 | |
| GC | 17.83 |  |  | SVs.MALY.DE | | 63.07 | | 1,574 | |
| CLLE.ES | 17.46 | 348,176 |  | SVs.CLLE.ES | | 51.39 | | 756 | |
| RepliTime | 16.51 |  |  | GC | | 8.97 | |  | |
| RecRate | 12.02 |  |  | Quadruplex | | 7.43 | |  | |
| Quadruplex | 11.78 |  |  | RepliTime | | 3.72 | |  | |
| Breakome | 9.34 |  |  | RecRate | | 3.39 | |  | |
| LAPs | 6.08 |  |  | CTCF | | 1.2 | |  | |
| PRDM9 | 3.81 |  |  | Breakome | | 0.5 | |  | |
| BOCA.FR | 0.62 | 36,374 |  | PRDM9 | | -0.62 | |  | |
| CTCF | -1.66 |  |  | LAPs | | -1.63 | |  | |
|  |  |  |  |  | |  | |  | |
|  |  |  |  |  | |  | |  | |

*6. As determined by the authors in a few instances, the LAP dataset may have a number of false negative for their analysis, either from insufficient Hi-C coverage, or from the lack of Hi-C data in an appropriate tissue. This likely confound their comparison, such as non-CTCF-LAP vs CTCF-LAP presented in fig 6. The authors need to provide a measurable sense of how the LAP definition impacts their findings. The authors could for example present results on different sets of LAPs with different level of confidence, generated from union, sample specific, or intersection from down-sampled Hi-C data.*

**Response:**

We have shown (Fig. S5) that LAP discovery is an approximately linear function of sequencing depth, based upon the Hi-C data of Rao et al (2014), and this is the justification for using the union of LAPs for most analyses. The false negatives the reviewer refers to are LAPs that could not be detected in the Hi-C data available. These false negatives can only occur at the CTCF sites *outside* annotated LAPs, and these CTCF sites do show some similarities to CTCF-sites inside LAPs, as we discuss in the manuscript. However, the non-CTCF LAP *versus* CTCF-LAP comparison mentioned by the reviewer should not pose any problem since both are defined conservatively, but we apologise if this has led to any confusion. The non-CTCF LAPs lack any evidence of binding CTCF since we conservatively excluded all LAPs that contain CTCF motifs or are bound by CTCF according to ChIP-seq data. These LAPs are compared to the CTCF-LAPs identified by Rao et al. to bind CTCF via properly orientated motifs. Hence, we do not think that false negatives are a problem in the non-CTCF-LAP vs CTCF LAP comparison, and this comparison crucially shows that CTCF alone does not lead to genomic instability at LAPs (Fig. 6b).

We argue that, since we find a LAP effect on mutation rates even in tissues where the LAPs were not sampled, this only shows that we are, if anything, underestimating the effect, i.e. our analysis is conservative with respect to any conclusions based on the union set of LAPs.

The figure below shows pan-cancer SNV rates at the seven original (cell-type specific) LAP datasets from Rao et al. Mutation rates clearly follow the same pattern, even using LAP datasets that are smaller and not tissue matched.

*7. In the association with BRCA1/2 binding in breast cancer, as the authors points out, active transcription can explain the association. Similar to the previous comments above, the authors need to provide more insight by using a regression-based method to control for transcription rate, or from the 15 ChromHMM type of regions derived by ENCODE/HMEC. Is their finding at LAPs somewhat independent or is their observation only a correlation with known factors?*

**Response:**

We thank the reviewer for this suggestion. As suggested, we have now added this analysis: We used the ChromHMM annotation for HMEC (human Mammary Epithelial Cells) to partition the genomic regions at LAPs into the 15 different states and ran a random forest analysis on the outcome variable “BRCA binding”. Indeed, the modelling suggests that BRCA1 and RAD51 binding is largely driven by chromatin context, rather than the presence of LAPs. The strongest influences on binding appear to be the presence of active promoters and enhancers. We have changed the text to make this clearer and add a Supplemental Table: “This is, to our knowledge, the first observation of BRCA1/2 association with LAPs, albeit that this appears to be driven by neighbouring active promoters and enhancers, rather than the LAPs themselves (Additional File 2: Table S1).”

In the Methods section, we have added this text under the heading “Random Forest regression analysis of BRCA1/2 binding”: “To model the outcome variables “BRCA1 binding” and “RAD51 binding” in MCF-10A and MCF-7 cell lines, respectively, we divided the genome up into 5kb windows around 1MB of HMEC LAPs. Input features to the model were all 15 Broad chromHMM states for state segmentation in HMEC cells as well as the overlap with HMEC LAPs. 200 random forest trees were constructed.”

*8. In figure 4B, the control by shifting by 50kb is interesting, and indeed likely corrects for replication timing and nuclear compartment. What is not clear however is the contribution of more local factors such as GC content, presence of PRDM9 or CTCF binding sites or motifs. Can the authors refine the observed association by looking at the contribution of known, tissue independent factors?* 

**Response:**

We are again grateful to the reviewer for prompting a re-examination of these data. On closer examination, we have found an intriguing difference in the local factors present at LAPs coinciding with recombination hotspots (HSs). In the revised manuscript, we raise the possibility that the increased recombination rates seen at LAPs are driven by CTCF binding and enrichments of PRDM9 motifs at CTCF LAPs: **“**Interestingly, the overlap between recombination HSs and LAPs is confined to LAPs that bind CTCF, and is not found for the stringently defined set of 2,102 non-CTCF LAPs. Only 11% of non-CTCF LAPs overlap HSs (not significant by circular permutations) and only 5% contain the PRDM9 motif, which is a significant depletion compared to expectation (*p* = 0.0044).”

In the discussion, we have also added this sentence: “This observation is consistent with an enrichment of recombination HSs at CTCF- LAPs (but not at non-CTCF LAPs), while the enrichment of PRDM9 motifs within CTCF-LAPs also suggests a sequence based mechanism.“

*9. Since LAPs have a higher rate of DSB, do the SNV in the CTCF match a particular signature, such as the APOBED signature seen around Kataegis sites. Can the authors comment whether they believe the higher rate of SNV in CTCF is likely a consequence of cell autonomous processes such as APOBEC or DNA repair?*

**Response:**

In the revised manuscript, we have added additional analyses of the mutational signatures present at CTCF-LAPs, non-CTCF LAPs and CTCF motifs. We find that CTCF-LAPs and non-CTCF LAPs show very similar signatures in each tumour type, consistent with our conclusion that the two types of LAPs suffer similar mutational burdens even though the mutational spectra observed vary between tumour types. Mutational spectra at CTCF motifs also differ among tumour types (as in Kaiser et al, 2016), but there is no indication that CTCF sites are highly mutable due to processes such as APOBEC, which would skew the signature towards particular signatures (such as signatures 2 and 13 from the COSMIC database: https://cancer.sanger.ac.uk/cell_lines/signatures). An important caveat is that the sample size of mutations at CTCF sites is sometimes very small (e.g. the BOCA-FR cohort only has 18 mutations in total at CTCF sites).

We have added this text to the Methods section:

“The R package SomaticSignatures [73] was used to calculate mutational signatures at union CTCF-LAPs, non-CTCF LAPS and CTCF motifs for each tissue type separately.”

We have also added this text to the Results section:

“With respect to mutation rates, the non-CTCF LAPs behave similarly to CTCF-containing LAPs and contain similar mutational signatures to CTCF-LAPs (Fig. 6 and Additional File 1: Figure S6), while CTCF motifs inside CTCF-LAPs showed no evidence for specific mutational signatures associated with processes such as APOBEC editing after single strand DNA exposure [53, 54] (Additional File 1: Figure S7).”

*10. The comparison between the three categories of LAPs presented in Fig5 A, is difficult to interpret as these 3 categories do not seem to be mutually exclusive. Wouldn't this analysis be more powerful and likely more interesting, if the observations were better contrasted in non-overlapping sets of LAPs?*

**Response:**

We thank the reviewer for this comment and have altered the LAPs presented in Fig. 5 to only show the mutually exclusive categories: “HSs”, “LAPs with HS overlap” and “LAPs without HS overlap”. This improves the clarity of the figure but our conclusions remain unchanged, i.e. HS-LAPs behave like LAPs with respect to cancer mutation rates, but more like HSs at the population polymorphism level.

We have also modified a sentence in the Results section to: “LAPs are associated with similar mutational landscapes in tumours, irrespective of whether they overlap HSs or locate outside high recombination regions: both types of LAP show a distinctive dip in tumour SNV rates (Fig. 5a)…”

*11. The striking differences between Cancer types presenting in Figure S7 is concerning. While a Pan-Cancer dataset increases statistical power, its analysis can be confounded when strong differences exist between the cohort sizes and mutational patterns as suggested in Table S1. The authors need to discuss how the differences may impact their observations and ideally take cancer type into consideration using a regression model.*

As indicated in our response above to point 4, we have now added a random forest regression analysis of the pan-cancer dataset, including cell type-invariant features only (GC content, recombination rate, average replication timing, constitutive CTCF binding site overlap, union LAP presence and quadruplex sequence overlap). The results of this analysis are presented in Table S3. We note that the influence of “LAP presence” on mutation rates does appear to vary between tumour types but is non-zero for all types (Figure S8; previously Figure S7).

As the reviewer suggests, one might expect to have seen large differences between tumour types attributable to the different mutational spectra and cohort sizes present. Instead, we see similar trends across cancers. For instance, breast tumours and melanomas show similar mutational profiles at LAPs (Figure S8) in spite of possessing very different mutational spectra (Figure S6). While breast and prostate tumours show similar profiles at LAPs in spite of very different cohort and mutational sample sizes. There are also tumour types (such as malignant lymphoma and chronic lymphocytic leukemia) that fail to show clear mutational patterns at LAPs. In this context, rather than ‘cherry picking’ particular tumour types, we have chosen to include all available mutational data and report the pan-cancer patterns. This is expected to dilute the patterns seen at LAPs over all samples and make our pan-cancer observations conservative. In parallel, we also exploit the largest cohort of breast tumours, along with matched chromatin data, to explore the pan-cancer patterns in more depth.

*12. In Fig6B, the authors claim the high fragility at non-LAP CTCF, but the data presented seems particularly noisy. Can the authors present a statistical evaluation to determine if the enrichment is significant?*

**Response:**

We confirmed that the 5KB windows around CTCF sites that are not assigned to any LAPs indeed show a statistically increased rate of breakage: we resampled the breakpoint rates at non-LAP CTCFs 1mio times with replacement, which suggests that the observed breakpoint rate in the central window (at the CTCF sites) is indeed higher than 99% of the resampled data. There is also a high rate of BRCA binding for these non-LAP CTCF sites, which is also indicative of DNA breakage. Accordingly, we have modified the text in the Results section:

“Non-LAP CTCF sites also showed an increased rate of DNA breakage (Fig. 6b) and a strong enrichment of BRCA1 (Additional File 1: Figure S4), consistent with general co-binding of these factors and recent data which suggest that CTCF has roles in regulating the homologous recombination repair pathway [52], as well as a high degree of genome fragility for these non-LAP CTCF sites.“

We added a description of the statistical test to the Figure legend of Fig.6: “The central 5kb windows of all three plots in (b) are statistically elevated compared to the flanking regions (resampling 1mio times with replacement; *p* < 0.01).**”**

*Minor Points

13. Despite a very comprehensive list of referenes, the autors missed important early reports documenting, likely for the first time, the association between copy number breakpoint and chromatin loops from Hi-C. (*[*https://www.ncbi.nlm.nih.gov/pmc/articles/PMC3460994/*](https://www.ncbi.nlm.nih.gov/pmc/articles/PMC3460994/) *and* [*https://www.ncbi.nlm.nih.gov/pubmed/22101486*](https://www.ncbi.nlm.nih.gov/pubmed/22101486)*). These references (and ref 16) may diminish some of the novelty of their findings regarding DSB enrichment at LAPs and their work should be discussed with these in perspective.*

**Response:**

We apologize for this oversight and now cite the two papers in the introduction as follows. “Further, the 3D structure of the genome may predispose regions of the genome that are in Hi-C contact to be more likely to undergo structural rearrangements [31, 32].”

Although these two papers have shown a possible association of DNA breakage and Hi-C contacts, we note that there are important differences from our study. Both papers operate on the megabase-level for their contact matrices and do not examine LAPs - in fact, LAPs had not been discovered at the time of these publications. Instead, they study the interaction preferences of large regions based on amalgamations of Hi-C interactions over megabase regions. They also use imprecise breakpoint datasets with much smaller sample sizes. Engreitz et al (2012) studied 1,533 translocations from cancer and normal genomes, while Fudenberg et al (2010) studied 39,568 copy number changes based upon low resolution array data. It was consequently not possible for either of these publications to discuss tumour mutation profiles at LAPs.

*14. In the title, and abstract (line 33) the authors state the association between LAPs and HS is high and unexpected, but the overlap is only 16%. Can the authors clarify whether the LAPs are significantly associated with HS and if not the case, edit the title and abstract?*  

**Response:**

We show in Fig. 4 that the overlap between LAPs and HSs is higher than expected and provide the p-value for this, based on circular permutations (p < 10^-5^).

*15. Line 61: Cohesion should be cohesin (autocorrect error)*

**Response:** We have changed this.

*16. The abbreviation of kilobase is kb no Kb.* 

**Response:**

We have changed this throughout the text.

*17. In Table1 why is the expected only for the triple and quadruple intersection? Can the authors provide numbers for the other comparisons? Can the authors provide the results of the statistical analysis either using hypergeometric test or the FDR permutation-based analysis?* 

**Response:**

The expected fractions were only shown for the triple and quadruple intersections since they were calculated based on the double intersection values, i.e. under feature independence, the probability of an intersection is the product of their respective probabilities. We have added the text below to the legend of Table 1 to make it clearer how the expected fractions were calculated, assuming independence of features. We now also added a *p*-value for these intersections, based on the hypergeometric test.

For completeness, we now also show the expected fractions of single overlaps (e.g. LAPs ∩ Boundaries) in Table 1b. This, however, was calculated using circular permutations since we ask a different question, i.e. do LAPs overlap more often with boundaries than expected, given their relative size and distribution in the genome.

“**a)** The number of features and their genomic span. **b)** Shown is the number of LAPs that overlap with domain boundaries, HSs or DSB-prone regions, respectively, as well as the fraction of unique LAPs in each intersection (as a proportion of the total dataset of 14,737 LAPs). The expected fraction (column 4) and the corresponding *p*-value (column 5) were calculated based on 10,000 circular permutations in *R*. **c)** Columns 2 and 3 as in (b). The expected fraction (column 4) was calculated by multiplying the respective fractions shown in column 3 of (b). For example, the fraction of LAPs in the intersection “LAPs ∩ HSs ∩ DSB-prone regions” is equal to 0.16 x 0.69 = 0.11. The hypergeometric test was used to assess the statistical significance of overlap (column 5).

*18. In contrast to their claim online 270, some of the Hi-C data should actually be fairly relevant. For example, GM12878 is a lymphoblastic cell line and may relate well to the CLL dataset. Similarly, NHEK may relate to Melanoma. Can the authors discuss more thoroughly why they thought HUVEC/Breast was a better histology to focus on?*

**Response:**

We did not focus on the CLL dataset since there are very few somatic mutations for this cancer type (see Table S2). Melanoma is a malignancy derived from melanocytes, which are quite different from (NHEK) keratinocytes. The HMEC cell line and the majority of breast tumours are all derived from breast epithelial cells; HMEC data is also widely used in other breast cancer studies.

*19. The details of the Gene-Set enrichment analysis should be presented in the method section (they are only briefly mentioned in the supplemental table header). The selection of the control dataset for enrichment as well as the gene set tested should be more explicit.*

**Response:**

We have now added the following text, under the new subheading “Functional enrichment analysis of recurrently disrupted LAPs” to the Methods section: “As the background set for enrichment analyses, we used the 9,973 genomic regions encompassed by all loops within the union set of LAPs. As the foreground set, we used 398 genomic regions encompassed by LAPs disrupted five or more times across tumour samples, corresponding to the top 5% most disrupted loops.”

*20. Line 317: clarify what recurrently disrupted mean in this context. Number of breast cancer donors?*

**Response:**

Yes, we changed the text to make this clearer: “In the breast cancer dataset, we also observe an unexpected excess of overlap between recurrently disrupted HMEC loops (bordered by LAPs that are disrupted in at least two samples) and GWAS regions associated with breast cancer”

*21. The method section needs to be more structured, so that the sub-sections related to specific analysis can be easily identified.* 

**Response:**

We have added subheadings to the methods section to make it easier to read and identify specific analyses.

**Responses to Reviewer #2**

*Reviewer #2: This is an interesting study of the association of LAPs with SNVs, SVs, chromatin marks, CTCF binding sites, and meiotic recombination hotspots in cancer versus germline cells. The conclusions are supported by the data. I have a few comments which would make the paper stronger.
Major:
1. Of the overlapping 16% LAPs and HSs, what percentage have PRDM9 core motifs?*

**Response:**

HS-overlapping LAPs are not more enriched for the PRDM9 motif than the complete set of LAPs. We have added this text the Results section: “For the 16% of LAPs directly overlapping HSs (HS-LAPs) we find no further enrichment of the PRDM9 motif (13.6% of HS-LAPs contain the motif)….”

However, as we point out above (response to point 8 of reviewer 1), we now also show that, in contrast to CTCF-containing LAPs, non-CTCF LAPs are not enriched for recombination HSs and add this text to the Results section:

**“**Interestingly, the overlap between recombination HSs and LAPs is confined to LAPs that bind CTCF, and is not found for the stringently defined set of 2,102 non-CTCF LAPs. Only 11% of non-CTCF LAPs overlap HSs (not significant by circular permutations) and only 5% contain the PRDM9 motif, which is a significant depletion compared to expectation (*p* = 0.0044).”

In the discussion, we have also added this sentence: “This observation is consistent with an enrichment of recombination HSs at CTCF- LAPs (but not at non-CTCF LAPs), while the enrichment of PRDM9 motifs within CTCF-LAPs also suggests a sequence based mechanism.“

*2. Are LAPs increased at cancer fragile gene sites (e.g. WWOX, FHIT, A2BP1)?*

**Response:**

We thank the reviewer for this question, which has yielded interesting results. To answer this question, we intersected the set of union LAPs and the coordinates of 70 protein-coding genes in fragile regions (annotated in the NCBI Gene database as “common fragile sites”). As a comparison, we also intersected the set of union LAPs with all protein-coding genes in the human genome. We found that, compared to all protein-coding genes, there are more intersections with LAPs per gene for fragile site genes. Since fragile site genes are interrupted more often by LAPs, we now argue that this is a mechanism that might contribute to their genomic instability.

We inserted the following text in the Methods section:” The genomic locations of 70 protein-coding genes within CFSs (annotated as “common fragile sites”) were downloaded from the NCBI Gene database.”

The following text was added to the Results section: “Common fragile sites (CFS) are large, initially cytogenetically defined, genomic regions, characterized by high rates of DNA breakage, active transcription and late replication [56, 57]. Interestingly, we found more LAP-gene intersections for protein-coding genes within CFSs compared to LAP-gene intersections in the genome as a whole (0.77 and 0.47 LAPs per gene; relative proportion test in R: p < 10^-6^), while the average replication timing was indistinguishable between the two sets of LAPs (Wilcoxon test in R: N.S.). If genes are more often interrupted by LAPs in fragile regions, this leaves the intriguing possibility that at least a subset of CFSs are caused by DNA looping and the instability associated with LAPs.”

*3. Are non-CTCF LAPs enriched for other binding motifs?*

**Response:**

Non-CTCF LAPs are overall less enriched for transcription factor motifs compared to CTCF-LAPs. We intersected both types of LAPs with the genome-wide set of 197,374 sites matching 118 motifs within constitutively open chromatin (and therefore putatively active binding sites) from Kaiser et al. (2016) and found that all of the 118 motifs were more enriched at CTCF LAPs compared to non-CTCF LAPs.

We added this text to the Results section: “The most common motifs at non-CTCF LAPs were the promoter-associated motifs MA0506.1 (NRF1), MA0516.1 (SP2) and MA0079.3 (SP1), but none of these motifs showed any enrichment compared to CTCF-binding LAPs.”

And to the Methods section: “To test for an enrichment of other transcription factor binding motifs at non-CTCF LAPs, we intersected the genomic coordinates of these LAPs with the locations of 118 motifs found in constitutively open chromatin [21]. “

*4. The Spearman values in Fig. 7C are extremely low, it's not clear whether these are meaningful.*

**Response:**

We agree that the individual correlations are modest, but the intention is simply to highlight that the trends for SNV and SV rates are in opposite directions. To clarify this, we have modified the relevant text in the Results section to: “However, even though the magnitude of effects is rather modest, the direction of associations is strikingly inverted for SNV and SV rates, such that the variables most strongly correlated with elevated SV rates (DNaseHS, replication timing, G-quadruplex content, GC content) are associated with decreased SNV rates (Fig. 7c).”

*5. It's not clear exactly how LAPs were defined/identified*

**Response:**

To make it clearer how the union set of LAPs was defined, we have added the following text to the Methods section: ” For this purpose, the seven files annotated as *looplist_with_motif* for human cell lines were downloaded from the GEO dataset GSE63525; the genomic locations of CTCF motifs assigned to LAPs were merged if overlapping, such that each motif was only counted once, and the flanking sequence extended to 5kb.”

Minor:
*1. Fig 1B missing labels of the different lines.*

**Response :**

The different colours of lines correspond to the two Y–axis labels, which are coloured in the same way. We made added the following text to the figure legend to clarify this: “(b) Finer scale average SNV rates per tumour (green line) within 1 kb of LAPs, centred on the CTCF site within each LAP, with nucleosome occupancy based on MNase-seq data over the same intervals (grey line).”

*2. Need to properly introduce the 1KG dataset before describing its results.*

**Response:**

We have replaced the acronym with the full name and inserted the citation to the 1KG project when we first mention it in the Results section. “As expected, recombination HSs are associated with a pronounced increase in SNPs in the 1000 Genomes Project (1KG) dataset [45]…”

**Second round of review**

**Reviewer 1**

The response from Kaiser et al is very satisfactory. They have properly addressed my comments and concerns and I appreciated the extra analysis provided, which rendered the report much clearer and relevant.
